# Assessing the impact of type 2 diabetes on mortality and life expectancy according to the number of risk factor targets achieved: an observational study

**DOI:** 10.1186/s12916-024-03343-w

**Published:** 2024-03-13

**Authors:** Bin Wang, Yanqi Fu, Xiao Tan, Ningjian Wang, Lu Qi, Yingli Lu

**Affiliations:** 1 Department of Endocrinology and Metabolism, Shanghai Ninth People’s Hospital, Shanghai Jiao Tong University School of Medicine, 639 Zhizaoju Road, Shanghai, 200011 China; 2 Department of Medical Sciences, Uppsala University, Uppsala, Sweden; 3 Department of Big Data in Health Science, School of Public Health, Zhejiang University School of Medicine, Hangzhou, China; 4 Department of Epidemiology, School of Public Health and Tropical Medicine, Tulane University, 1440 Canal Street, Suite 1724, New Orleans, LA 70112 USA; 5 Department of Nutrition, Harvard T.H. Chan School of Public Health, Boston, MA USA

**Keywords:** Risk factor, Type 2 diabetes, Mortality, Life expectancy, Cohort study

## Abstract

**Background:**

Type 2 diabetes (T2D) is associated with an increased risk of premature death. Whether multifactorial risk factor modification could attenuate T2D-related excess risk of death is unclear. We aimed to examine the association of risk factor target achievement with mortality and life expectancy among patients with T2D, compared with individuals without diabetes.

**Methods:**

In this longitudinal cohort study, we included 316 995 participants (14 162 with T2D and 302 833 without T2D) free from cardiovascular disease (CVD) or cancer at baseline between 2006 and 2010 from the UK Biobank. Participants with T2D were categorised according to the number of risk factors within target range (non-smoking, being physically active, healthy diet, guideline-recommended levels of glycated haemoglobin, body mass index, blood pressure, and total cholesterol). Survival models were applied to calculate hazard ratios (HRs) for mortality and predict life expectancy differences.

**Results:**

Over a median follow-up of 13.8 (IQR 13.1–14.4) years, deaths occurred among 2105 (14.9%) participants with T2D and 18 505 (6.1%) participants without T2D. Compared with participants without T2D (death rate per 1000 person-years 4.51 [95% CI 4.44 to 4.57]), the risk of all-cause mortality among those with T2D decreased stepwise with an increasing number of risk factors within target range (0–1 risk factor target achieved: absolute rate difference per 1000 person-years 7.34 [4.91 to 9.78], HR 2.70 [2.25 to 3.25]; 6–7 risk factors target achieved: absolute rate difference per 1000 person-years 0.68 [-0.62 to 1.99], HR 1.16 [0.93 to 1.43]). A similar pattern was observed for CVD and cancer mortality. The association between risk factors target achievement and all-cause mortality was more prominent among participants younger than 60 years than those 60 years or older (*P* for interaction = 0.012). At age 50 years, participants with T2D who had 0–1 and 6–7 risk factors within target range had an average 7.67 (95% CI 6.15 to 9.19) and 0.99 (-0.59 to 2.56) reduced years of life expectancy, respectively, compared with those without T2D.

**Conclusions:**

Individuals with T2D who achieved multiple risk factor targets had no significant excess mortality risk or reduction in life expectancy than those without diabetes. Early interventions aiming to promote risk factor modification could translate into improved long-term survival for patients with T2D.

**Supplementary Information:**

The online version contains supplementary material available at 10.1186/s12916-024-03343-w.

## Background

Type 2 diabetes (T2D) is a global public health challenge, with approximately 537 million adults living with diabetes worldwide in 2021 [1]. Patients with T2D are susceptible to developing macro- and micro-vascular complications that eventually lead to high rates of mortality [2, 3]. The risk of premature death in patients with T2D is about 2 times greater than in the general population [4]. Current treatment of T2D comprises continuous medical care with comprehensive strategies for improving adverse outcomes, and modifiable risk factors are typically outlined as primary targets [5].

Results from randomized trials support a beneficial effect of multiple risk factor interventions on improving cardiovascular health and survival among patients with T2D [6, 7]. Observational studies thus far have largely focused on associations between multifactorial risk factor modification and cardiovascular disease (CVD) [8–10], and only one prospective study has shown that patients with T2D who had all five risk factors (smoking, hemoglobin A_1c_ [HbA_1c_], blood pressure, cholesterol, and albuminuria) within target range exhibited a marginally higher risk of death than the general population [10]. Given that smoking was the single lifestyle factor in this study, whether achieving targets for other lifestyle factors such as physical activity and diet, which have been individually associated with all-cause mortality in T2D, might eliminate the residual elevated risk of death warrants further investigation [11]. Furthermore, in comparison with relative risk, life expectancy as an absolute quantitative measure for premature mortality is more intuitive regarding public health implication. However, the extent to which life expectancy loss associated with T2D could be offset by multiple risk factor modification is unclear.

To fill the critical knowledge gap, we quantified all-cause and cause-specific mortality risk among patients with T2D, according to the number of risk factors within the guideline-recommended target range, as compared with individuals without diabetes from the large UK Biobank cohort. We also evaluated the association between the degree of risk factor target achievement and life expectancy in the T2D population. Identifying these features could help tailor appropriate interventions to reduce T2D-related mortality risk.

## Methods

### Study design and population

The UK Biobank is a prospective, population-based cohort of more than 500 000 participants aged 40–69 years who were recruited from 22 assessment centres across the UK during 2006–2010. Participants completed touchscreen questionnaires, underwent physical measurements, and provided biological samples, as described in detail previously [12]. The UK Biobank study received ethics approval from the North West Multi-Center Research Ethics Committee (reference no. 11/NW/03820). All participants gave written informed consent.

For this study, participants with T2D at baseline were identified through hospital inpatient records and a validated algorithm based on self-reported medical history and medication information [13]. Individuals without a previous diagnosis of diabetes but had blood glucose levels above the T2D threshold, defined as random glucose ≥ 11.1 mmol/L or HbA_1c_ ≥ 48 mmol/mol (≥ 6.5%) according to the American Diabetes Association 2010 criteria [14], were defined as undiagnosed cases and assumed to have T2D. The cohort of participants with and without T2D was constructed after exclusion of those with prevalent CVD or cancer at baseline, type 1 diabetes, or missing values of exposure, or those who had withdrawn from the UK Biobank. Prevalent diagnoses were determined via a combination of self-report and hospital inpatient records (Additional file 1: Table S1).

### Data collection

Information on sociodemographic status, lifestyle, history of medical conditions, and medication use was collected through touchscreen questionnaires or verbal interviews at baseline. Area-based socioeconomic status was derived from the postcode of residence using the Townsend deprivation index (TDI), with a lower score indicating a higher level of socioeconomic status [15]. Smoking status was self-reported and categorised as never, former, or current smoker. Physical activity was assessed using the International Physical Activity Questionnaire short form. The habitual consumption of a range of common food items was obtained from the food frequency questionnaire. Physical measurements, including height, weight, and blood pressure, were performed by nurses at baseline. Body mass index (BMI) was calculated as weight in kilograms divided by the square of height in meters. Blood pressure was measured twice for each individual and the mean of two readings was used. The standard protocols for measurements of biochemical markers have been described elsewhere [16], including HbA_1c_ (VARIANT II Turbo Hemoglobin Testing System, Bio-Rad), and serum triglycerides and cholesterol (AU5800 clinical chemistry analyser, Beckman Coulter). Diabetes duration was calculated from the date of diabetes diagnosis to baseline assessment for diagnosed patients and assumed 0 years for undiagnosed patients.

### Assessment of risk factors

Risk factors were assessed in participants with and without T2D. We selected seven risk factors based on the recommendations from the American Heart Association Task Force and the National Institute for Health and Care Excellence guidance in the UK [17, 18], and defined whether these risk factors were within target range using the following guideline-recommended target levels: no current smoking, physical activity at goal (≥ 150 min/week of moderate activity or ≥ 75 min/week of vigorous activity or an equivalent combination), healthy diet (adherence to at least four of the seven endorsed characteristics—increased consumption of fruits, vegetables, whole grains, and fish and reduced consumption of refined grains, processed meat, and unprocessed meat) [19], BMI ≥ 20 and < 25 kg/m^2^, systolic blood pressure < 140 mmHg and diastolic blood pressure < 90 mmHg, HbA_1c_ < 53 mmol/mol (< 7%), and total cholesterol < 5.2 mmol/L (< 200 mg/dL). A detailed definition of risk factors can be found in Additional file 1: Table S2. We did not consider low-density lipoprotein (LDL) cholesterol as a risk factor because evident evidence has shown that low LDL cholesterol levels are associated with increased mortality risk in the general population and patients with T2D [10, 20–22]. In a recent study of the UK Biobank, a significant U-shaped relationship was also observed between LDL cholesterol and all-cause mortality [23].

### Ascertainment of outcomes

The outcomes in the present study were all-cause mortality and mortality from CVD and cancer. The date and cause of death were obtained from the death certificates of the National Health Service Information Center (England and Wales) and the National Health Service Central Register (Scotland). Detailed information about the linkage procedure is available online (https://content.digital.nhs.uk/services). Cause-specific mortality was ascertained using the International Classification of Diseases tenth Revision codes: I00-I99 for CVD mortality and C00-C97 for cancer mortality (Additional file 1: Table S1). At the time of analysis, person-time was censored at the date of death or the date of available death datasets (19 December 2022), whichever occurred first.

### Statistical analyses

For the main analysis, we estimated the risk of all-cause and cause-specific mortality among participants with T2D according to the number of risk factors within target range compared with participants without T2D. Because of the limited T2D cases in extreme categories, the number of risk factors within target range were grouped into 6 categories (0–1, 2, 3, 4, 5, and 6–7). Cox proportional hazards regression with age as the time scale was used to calculate hazard ratios (HRs) and 95% CIs for mortality. The proportional hazards assumption was verified using the Schoenfeld residuals method. All analyses were stratified on birth cohort (5 year groups) to take into account differences in baseline hazard due to birth year and adjusted for sex, ethnicity, education, and TDI. Missing covariate data were coded as a missing indicator category for categorical variables. To assess whether the pattern of association varied by age and sex, we tested interaction terms with age and sex in the adjusted models. We used Poisson regression to calculate covariate adjusted incidence rates and absolute rate differences per 1000 person-years and 95% CIs.

Among participants with T2D, we estimated the mortality risk in relation to each risk factor and the number of risk factors within target range with additional adjustment for diabetes duration. For analyses on individual risk factors, all risk factors were simultaneously included in the models. We performed similar analyses among participants without T2D and tested interactions between the presence of T2D and the number of risk factor targets achieved.

The calculation of differences in life expectancy (i.e. years of life lost) associated with risk factors involved a two-step process using flexible parametric survival models. First, residual life expectancy was estimated as the area under the survival curve from age 45 up to 100 years (1-year intervals) for participants without T2D and those with T2D following different number of risk factor within target range. Second, years of life lost were calculated as the difference in life expectancy at any given age between participants without T2D and each of the risk-factor groups for those with T2D [24]. Flexible parametric survival models were run with the stpm2 command which uses restricted cubic splines to model the baseline cumulative hazard [25, 26].

Several sensitivity analyses were conducted to test the robustness of our results. First, analyses were repeated with alternative definitions of risk factors, including use of different cutoff values for HbA_1c_ (< 48 mmol/mol [< 6.5%]), blood pressure (< 130/80 mm Hg), and BMI (≥ 20 and ≤ 30 kg/m^2^), inclusion of sleep duration (cutoff 7–8 h/day) as an additional risk factor, and consecutive exclusion of each of the seven risk factors. Second, we additionally adjusted for prevalent morbidity and estimated glomerular filtration rate. Third, we repeated the analyses by redefining the reference group as individuals without T2D who had different number (0–3, 4 [median number], or 5–7) of risk factors within target range. Fourth, we constructed a weighted score of risk factors considering the effect size for individual factors derived from the adjusted model. Fifth, to evaluate the influence of missing data, multiple imputation by chained equations was used to assign missing values for risk factors and covariates. We imputed five complete data sets and the variables that were used in the imputation model included all risk factors, selected covariates for adjustment, diabetes status, medications, and assessment center. The estimates from each imputed data set were pooled into one overall estimate with the use of Rubin’s rule. Sixth, we accounted for competing events by applying Fine and Gray proportional subdistribution hazards regression for cause-specific mortality. Seventh, models were rerun after excluding participants who died within the first 2 years of follow-up to reduce potential reverse causality. Finally, patients with undiagnosed T2D were excluded in case lifestyle might drastically change after the awareness of diabetes.

All statistical analyses were performed with SAS (version 9.4) and Stata (version 14). A two-sided *P* value < 0.05 was considered statistically significant.

## Results

The study population included 316 995 participants (14 162 with T2D and 302 833 without T2D) with complete data on all seven risk factors (Additional file 1: Fig. S1). The baseline median age was 57 (IQR 49–62) years and 171 138 (54.0%) were women. Of the 14 162 participants with T2D, 594 (4.2%), 2106 (14.9%), 3947 (27.9%), 4122 (29.1%), 2507 (17.7%), and 886 (6.3%) had 0–1, 2, 3, 4, 5, and 6–7 risk factors within target range, respectively. Individuals with a higher number of risk factors within target range were more likely to be women, non-White, and have a higher education and lower socioeconomic deprivation (Table 1). The number of risk factors within target range was generally higher in participants without T2D than in those with T2D (Additional file 1: Fig. S2).

**Table 1:** Baseline characteristics of participants with and without type 2 diabetes

	**Individuals without diabetes**	**Individuals with type 2 diabetes**						
		**Overall**	**No. of risk factors within target range**^a^					
			**0–1**	**2**	**3**	**4**	**5**	**6–7**
Participants	302 833	14 162	594	2106	3947	4122	2507	886
Age, years	57 (49–62)	60 (54–65)	58 (51–63)	59 (53–64)	60 (54–65)	61 (55–65)	61 (55–65)	61 (54–65)
Sex								
Female	165 553 (54.7)	5585 (39.4)	184 (31.0)	774 (36.7)	1437 (36.4)	1683 (40.8)	1103 (44.0)	404 (45.6)
Male	137 280 (45.3)	8577 (60.6)	410 (69.0)	1332 (63.2)	2510 (63.6)	2439 (59.2)	1404 (56.0)	482 (54.4)
Ethnicity								
White	289 584 (95.6)	12 454 (87.9)	531 (89.4)	1871 (88.8)	3543 (89.8)	3582 (86.9)	2197 (87.6)	730 (82.4)
Other	12 369 (4.1)	1652 (11.7)	62 (10.4)	224 (10.6)	395 (10.0)	525 (12.7)	296 (11.8)	150 (16.9)
Unknown	880 (0.3)	56 (0.4)	1 (0.2)	11 (0.5)	9 (0.2)	15 (0.4)	14 (0.6)	6 (0.7)
Education								
College or university degree	106 261 (35.1)	3711 (26.2)	121 (20.4)	518 (24.6)	960 (24.3)	1100 (26.7)	700 (27.9)	312 (35.2)
Other	194 629 (64.3)	10 284 (72.6)	462 (77.8)	1555 (73.8)	2943 (74.6)	2981 (72.3)	1778 (70.9)	565 (63.8)
Unknown	1943 (0.6)	167 (1.2)	11 (1.8)	33 (1.6)	44 (1.1)	41 (1.0)	29 (1.2)	9 (1.0)
Townsend deprivation index	-1.5 (2.9)	-0.7 (3.3)	-0.3 (3.3)	-0.6 (3.4)	-0.7 (3.3)	-0.7 (3.3)	-1.0 (3.2)	-0.9 (3.3)
Duration of diabetes, years^b^		2.7 (0.1–6.0)	0 (0–4.0)	2.0 (0.0–5.7)	2.8 (0–6.0)	3.0 (1.0–6.0)	3.0 (0.9–6.0)	3.0 (1.0–6.0)
Treatment								
Antihypertensive agents	45 487 (15.0)	7518 (53.1)	220 (37.0)	1014 (48.1)	2194 (55.6)	2306 (55.9)	1380 (55.0)	404 (45.6)
Lipid-lowering agents	30 655 (10.1)	8845 (62.5)	217 (36.5)	1066 (50.6)	2426 (61.5)	2746 (66.6)	1758 (70.1)	632 (71.3)
Oral antidiabetic agents		7405 (52.3)	213 (35.9)	1025 (48.7)	2117 (53.6)	2234 (54.2)	1342 (53.5)	474 (53.5)
Insulin		1325 (9.4)	53 (8.9)	214 (10.2)	403 (10.2)	389 (9.4)	194 (7.7)	72 (8.1)
No current smoking	272 655 (90.0)	12 653 (89.3)	316 (53.2)	1670 (79.3)	3494 (88.5)	3869 (93.9)	2428 (96.8)	876 (98.9)
Physical activity at goal^c^	165 582 (54.7)	6547 (46.2)	36 (6.1)	379 (18.0)	1312 (33.2)	2194 (53.2)	1820 (72.6)	806 (91.0)
Healthy diet^d^	127 665 (42.2)	5571 (39.3)	21 (3.5)	243 (11.5)	967 (24.5)	1844 (44.7)	1702 (67.9)	794 (89.6)
BMI, kg/m^2^	27.0 (4.5)	31.3 (5.8)	32.8 (5.6)	32.7 (5.7)	32.2 (5.6)	31.2 (5.6)	30.0 (5.6)	27.6 (5.6)
BMI ≥ 20 and < 25 kg/m^2^	100 475 (33.2)	1513 (10.7)	6 (1.0)	32 (1.5)	164 (4.2)	403 (9.8)	486 (19.4)	422 (47.6)
Systolic blood pressure, mm Hg	137.3 (18.5)	142.3 (17.2)	153.6 (16.5)	149.5 (16.2)	145.5 (16.7)	140.5 (16.6)	135.7 (15.9)	129.5 (12.0)
Diastolic blood pressure, mm Hg	82.4 (10.1)	83.3 (9.7)	91.1 (9.7)	87.6 (9.7)	84.9 (9.4)	82.2 (9.1)	79.5 (8.7)	76.8 (7.2)
Blood pressure < 140/90 mmHg	168 240 (55.6)	6123 (43.2)	27 (4.5)	327 (15.5)	1234 (31.3)	2014 (48.9)	1712 (68.3)	809 (91.3)
HbA_1c_, mmol/mol	34.8 (3.7)	52.2 (14.6)	65.9 (15.6)	59.6 (16.8)	54.3 (15.1)	49.6 (12.1)	46.5 (11.1)	43.9 (7.0)
HbA_1c_, %	5.3 (0.3)	6.9 (1.3)	8.2 (1.4)	7.6 (1.5)	7.1 (1.4)	6.7 (1.1)	6.4 (1.0)	6.2 (0.6)
HbA_1c_ < 53 mmol/mol (< 7%)	302 833 (100.0)	8924 (63.0)	61 (10.3)	676 (32.1)	2150 (54.5)	2996 (72.7)	2182 (87.0)	859 (96.9)
Total cholesterol, mmol/L	5.8 (1.1)	4.8 (1.2)	6.0 (1.2)	5.3 (1.3)	4.9 (1.2)	4.6 (1.1)	4.4 (0.9)	4.2 (0.8)
Total cholesterol < 5.2 mmol/L	89 407 (29.5)	9690 (68.4)	76 (12.8)	885 (42.0)	2520 (63.8)	3168 (76.9)	2205 (87.9)	836 (94.4)

Data are n (%), mean (SD), or median (IQR)

^a^Seven risk factors that were within target range include no current smoking, physical activity at goal, healthy diet, BMI ≥ 20 and < 25 kg/m^2^, blood pressure < 140/90 mmHg, HbA_1c_ < 53 mmol/mol (< 7%), and total cholesterol < 5.2 mmol/L (< 200 mg/dL)

^b^Diabetes duration was assigned as 0 years for undiagnosed patients

^c^Physical activity at goal was defined as ≥ 150 min/week of moderate activity or ≥ 75 min/week of vigorous activity, or an equivalent combination

^d^Healthy diet was defined as adequate intake of at least 4 of 7 recommended food groups

During a median follow-up of 13.8 years (IQR 13.1–14.4, 4.29 million person-years), 20 610 deaths were documented, with 3902 deaths from CVD and 10 205 deaths from cancer. A total of 2105 (14.9%) participants with T2D and 18 505 (6.1%) participants without T2D died during the study period. Compared with participants without T2D, those with T2D had an increased risk of all-cause mortality (HR 1.60 [95% CI 1.53–1.68]), CVD mortality (HR 1.78 [1.61–1.96]), and cancer mortality (HR 1.32 [1.22–1.42]) (Additional file 1: Table S3).

**Fig. 1:**
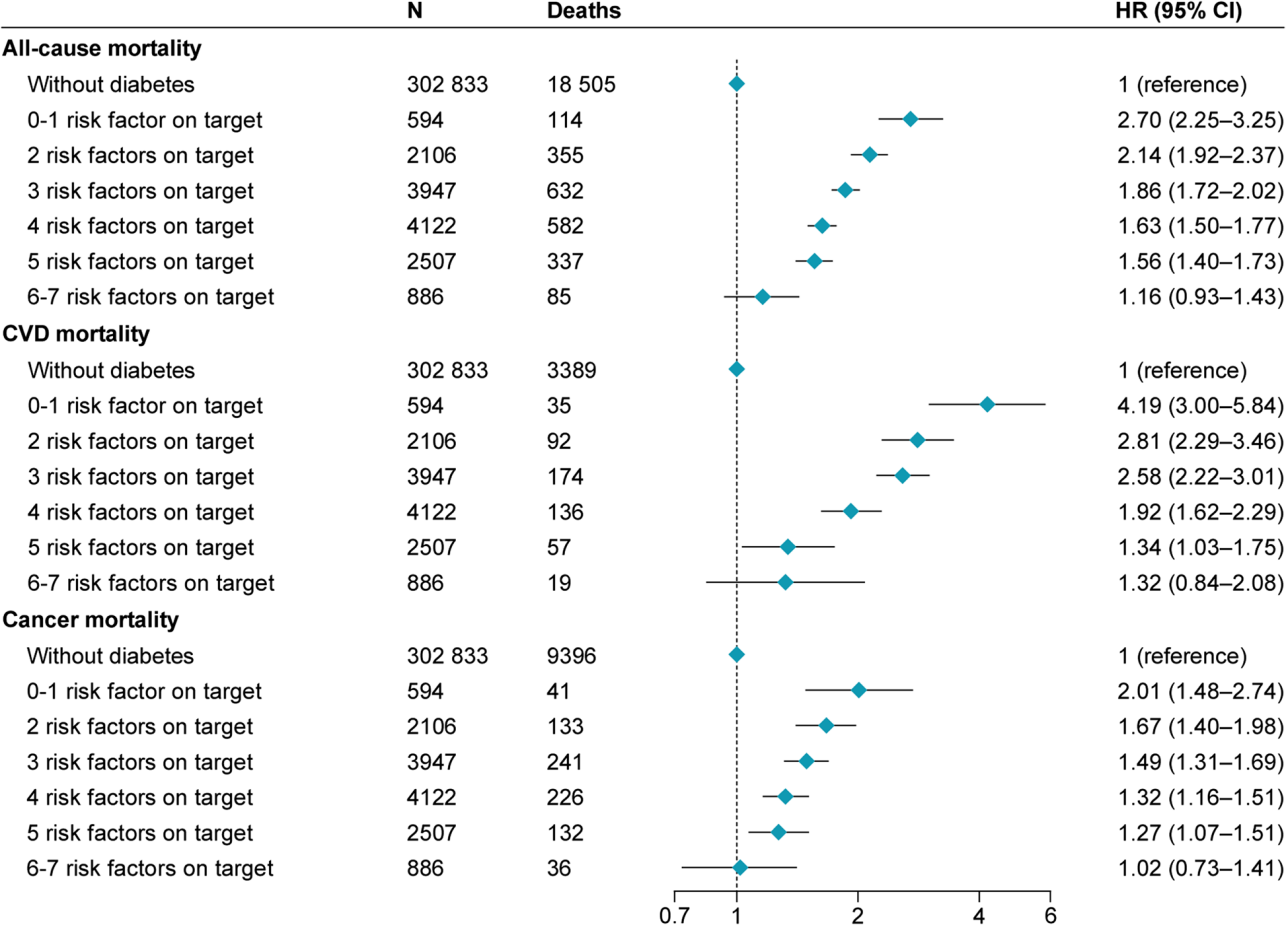
Risk of mortality according to the number of risk factors within target range among participants with type 2 diabetes compared with those without diabetes. Hazard ratio was estimated using Cox proportional hazards regression with age as time scale and adjusted for sex, ethnicity, education, and Townsend deprivation index. CVD, cardiovascular disease; HR, hazard ratio

As shown in Table 2 and Additional file 1: Fig. S3, the cumulative rate of mortality among participants with T2D decreased stepwise with an increasing number of risk factors within target range. Compared with the death rate of 4.51 (95% CI 4.44–4.57) per 1000 person-years among participants without T2D, the absolute rate differences were 7.34 (4.91 to 9.78) and 0.68 (-0.62 to 1.99) for participants with T2D who had 0–1 and 6–7 risk factors within target range, respectively. In multivariable Cox models, compared with participants without T2D, the HR for all-cause mortality among participants with T2D gradually decreased from 2.70 (95% CI 2.25–3.25) in those with 0–1 risk factor within target range to 1.16 (0.93–1.43) in those with 6–7 risk factors within target range (Fig. 1). A similar pattern of association was observed for CVD mortality (0–1 risk factor on target: HR 4.19 [3.00–5.84]; 6–7 risk factors on target: HR 1.32 [0.84–2.08]) and cancer mortality (0–1 risk factor on target: HR 2.01 [1.48–2.74]; 6–7 risk factors on target: HR 1.02 [0.73–1.41]).

**Table 2:** Rate for mortality among participants without diabetes and participants with type 2 diabetes according to the number of risk factors within target range

	**Individuals without diabetes**	**No. of risk factors within target range among individuals with diabetes**					
		**0–1**	**2**	**3**	**4**	**5**	**6–7**
**All-cause mortality**							
Deaths/person-years	18 505/4 107 161	114/7679	355/27 489	632/51 454	582/54 140	337/32 868	85/11 758
Death rate (95% CI)^a^	4.51 (4.44–4.57)	11.9 (9.65–14.6)	9.46 (8.38–10.7)	8.30 (7.55–9.12)	7.30 (6.61–8.05)	7.03 (6.18–8.00)	5.19 (4.04–6.67)
Rate difference (95% CI)^a^	0 (reference)	7.34 (4.91–9.78)	4.95 (3.80–6.10)	3.79 (3.00–4.58)	2.79 (2.07–3.51)	2.52 (1.61–3.43)	0.68 (-0.62 to 1.99)
**CVD mortality**							
Deaths/person-years	3389/4 107 161	35/7679	92/27 489	174/51 454	136/54 140	57/32 868	19/11 758
Death rate (95% CI)	0.83 (0.80–0.85)	3.39 (2.31–4.97)	2.26 (1.76–2.89)	2.10 (1.74–2.54)	1.59 (1.29–1.96)	1.13 (0.82–1.55)	1.11 (0.64–1.90)
Rate difference (95% CI)	0 (reference)	2.56 (1.26–3.86)	1.43 (0.87–1.99)	1.27 (0.88–1.67)	0.76 (0.43–1.10)	0.30 (-0.06 to 0.66)	0.28 (-0.32 to 0.88)
**Cancer mortality**							
Deaths/person-years	9396/4 107 161	41/7679	133/27 489	241/51 454	226/54 140	132/32 868	36/11 758
Death rate (95% CI)	2.29 (2.24–2.33)	4.56 (3.27–6.35)	3.78 (3.12–4.59)	3.38 (2.92–3.92)	3.01 (2.58–3.51)	2.92 (2.39–3.57)	2.30 (1.58–3.35)
Rate difference (95% CI)	0 (reference)	2.27 (0.76–3.78)	1.50 (0.77–2.22)	1.09 (0.59–1.60)	0.72 (0.26–1.19)	0.63 (0.05–1.22)	0.01 (-0.86 to 0.88)

Seven risk factors that were within target range include no current smoking, physical activity at goal, healthy diet, BMI ≥ 20 and < 25 kg/m^2^, blood pressure < 140/90 mmHg, HbA_1c_ < 53 mmol/mol (< 7%), and total cholesterol < 5.2 mmol/L (< 200 mg/dL)

^a^Death rate adjusted for age, sex, ethnicity, education, and Townsend deprivation index and absolute rate difference per 1000 person-years

CVD, cardiovascular disease

**Fig. 2:**
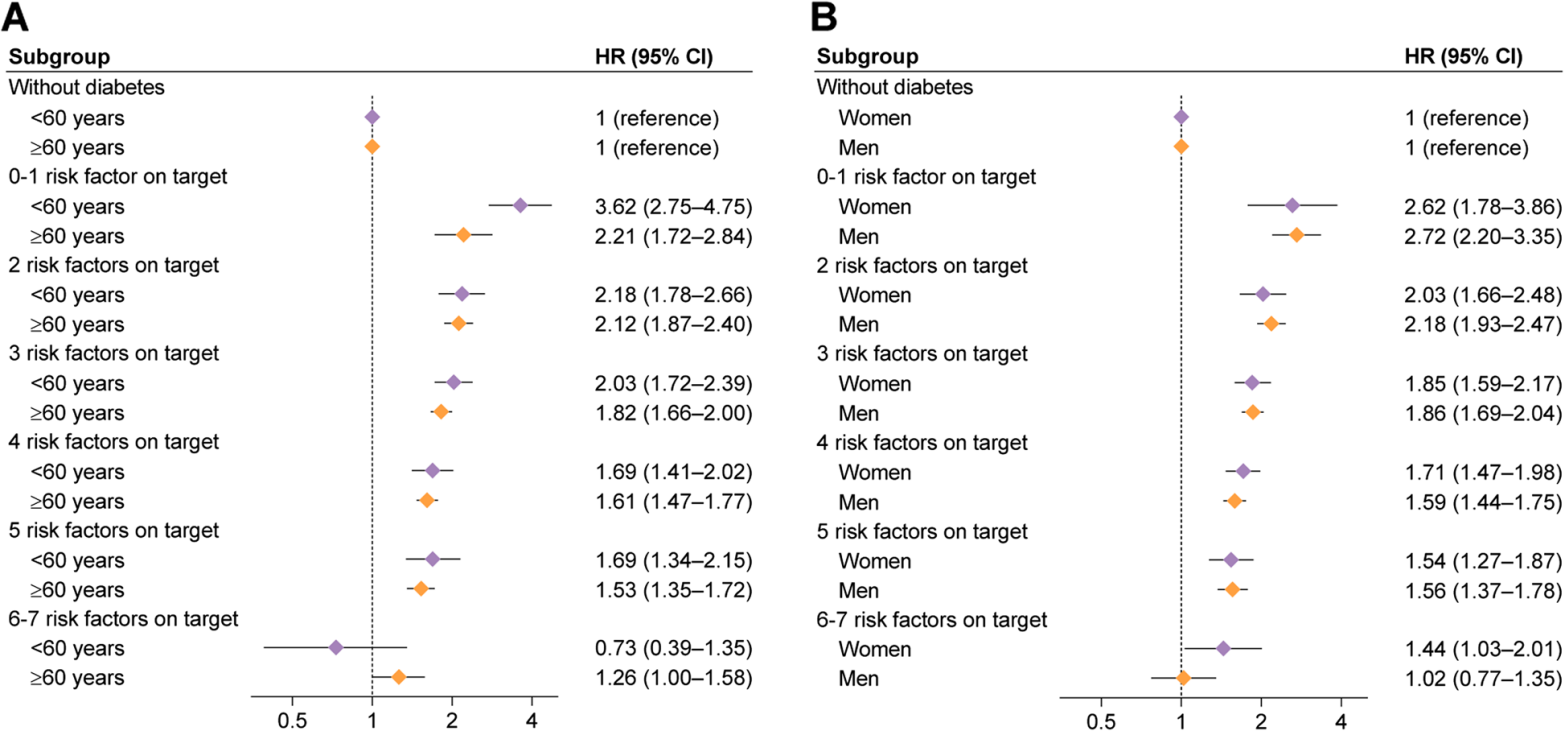
Risk of all-cause mortality according to the number of risk factors within target range among participants with type 2 diabetes compared with those without diabetes by age (**A**) and sex (**B**) categories. Hazard ratio was estimated using Cox proportional hazards regression with age as time scale and adjusted for sex (for analyses by age categories only), ethnicity, education, and Townsend deprivation index. Interactions with age and sex for the association of all-cause mortality according to the number of risk factors within target range among participants with diabetes compared to those without diabetes: *P* for interaction with age = 0.012; *P* for interaction with sex = 0.74. HR, hazard ratio

The gradual reduction in the excess risk of all-cause mortality associated with more risk factors within target range was greater among participants with T2D aged younger than 60 years (0–1 risk factor on target: HR 3.62 [95% CI 2.75–4.75]; 6–7 risk factors on target: HR 0.73 [0.39–1.35]) than those aged 60 years or older (0–1 risk factor on target: HR 2.21 [1.72–2.84]; 6–7 risk factors on target: HR 1.26 [1.00–1.58], *P* for interaction = 0.012; Fig. 2A). No significant interaction was observed with sex, even though the risk estimate of all-cause mortality for participants with T2D who had 6–7 risk factors within target range was attenuated toward the null in men only (Fig. 2B).

Among participants with T2D, individual risk factors showed an association with mortality with the exception of total cholesterol (Additional file 1: Table S4). For each 1-number increment in risk factors within target range, the HR was 0.88 (95% CI 0.85–0.91) for all-cause mortality, 0.79 (0.74–0.85) for CVD mortality, and 0.90 (0.85–0.95) for cancer mortality; the graded associations of more risk factor targets achieved with lower mortality risk were comparable between participants with and without T2D (Additional file 1: Table S5). In subgroup analysis among participants with T2D, consistent results were observed in strata of sex, education attainment, diabetes duration, and diabetes medication use (Additional file 1: Table S6). We observed interactions of risk factors with age and TDI on all-cause mortality and cancer mortality, whereby the HR estimates per additional risk factor within target range were more pronounced among those younger than 60 years and those with high TDI (*P* for interaction < 0.05).

**Fig. 3:**
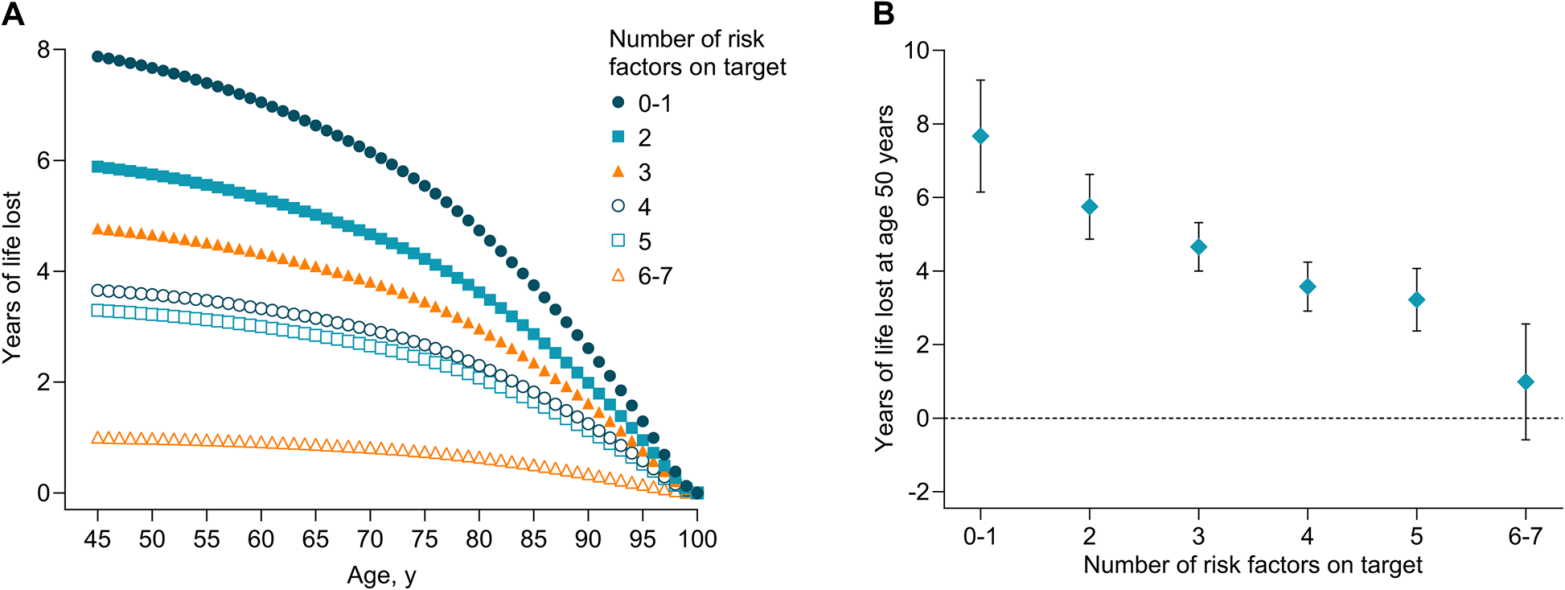
The estimates of years of life lost by the number of risk factors within target range among participants with type 2 diabetes compared with those without diabetes (**A**) Age-specific life-year lost by the number of risk factors on target among participants with type 2 diabetes versus those without diabetes. (**B**) Years of life lost at age 50 years by the number of risk factors on target among participants with type 2 diabetes versus those without diabetes. Models with age as time scale were adjusted for sex, ethnicity, education, and Townsend deprivation index

We estimated the differences in life expectancy associated with T2D according to different number of risk factors within target range. Compared with participants without T2D, the life expectancy at age 50 years was on average 7.67 (95% CI 6.15 to 9.19) years lower for participants with T2D who had 0–1 risk factor within target range and 0.99 (-0.59 to 2.56) year lower for those who had 6–7 risk factors within target range (Fig. 3A-B). Among participants with T2D, relative to those with 0–1 risk factor within target range, the life expectancy at age 50 years increased by an average of 1.30 (0.32–2.28) years for each additional risk factor target achieved (Additional file 1: Table S7).

Sensitivity analyses with a series of alternative definitions of risk factors or further adjustment for estimated glomerular filtration rate and prevalent morbidity yielded consistent results with the main analysis (Additional file 1: Table S8). Results were similar when the reference group was defined as participants without.

T2D with different number of risk factors within target range (Additional file 1: Table S9). In additional analyses by adopting weighted score of risk factors, using multiple imputation for missing values of risk factors and covariates, accounting for competing risk of cause-specific mortality, excluding participants who died during the first 2 years after recruitment, or restricting patients with T2D to diagnosed cases, the results did not substantially change (Additional file 1: Tables S10-S14).

## Discussion

In this prospective cohort analysis based on the UK Biobank, the mortality risk among participants with T2D decreased gradually with an increasing number of risk factors within the recommended target range. Participants with T2D who had 6–7 risk factors within target range had no significant excess risk of all-cause mortality and mortality from CVD and cancer compared with those without diabetes. The estimated life expectancy at age 50 years among participants with T2D was on average 7.7 years lower in those with 0–1 risk factor within target range and 0.99 year lower in those with 6–7 risk factors within target range than participants without diabetes.

To the best of our knowledge, this is the first study to specifically quantify the association between comprehensive risk factor control and all-cause and cause-specific mortality among patients with T2D. Randomised trials evaluating the effects of multifactorial interventions in T2D with death as the outcome are sparse and yield mixed results [6, 27–29]. In the Steno-2 trial among 160 patients with T2D and microalbuminuria, intensive cardiovascular risk factor intervention involving behaviour modification and pharmacologic treatment could halve mortality risk after a follow-up of 7.8 years [6]. In an extended follow-up of the cohort, life expectancy was up to 8 years longer for the intervention group [30]. By contrast, results from Look AHEAD (Action for Health in Diabetes) trial among 5145 overweight or obese patients with T2D showed that an intensive lifestyle intervention targeting weight loss did not reduce the rate of all-cause or cardiovascular mortality over a median follow-up of 9.6 years [29]. Observational studies with regard to survival among patients with T2D have mainly assessed lifestyle behaviours [11, 31–33], while data linking multiple risk factor modification with mortality are scarce. In the current study, we analysed a comprehensive risk factor profile by incorporating a total of seven lifestyle and metabolic risk factors to ascertain its association with mortality. Importantly, we illustrated that having 6 or more risk factors within target range was associated with no excess risk of all-cause, CVD and cancer mortality among patients with T2D when compared with individuals without diabetes. Our results were similar to those from a large Swedish Diabetes Register study, in which patients with T2D who had all 5 risk factors on target (no smoking and recommended levels of HbA_1c_, LDL cholesterol, blood pressure, and albuminuria) showed marginally higher risk of all-cause mortality than the general population [10]. Given the global epidemic of T2D and associated higher mortality risk, our findings emphasise the importance of promoting risk factor modification strategies to facilitate survival for patients with T2D.

Consistent with the results from the Swedish Diabetes Register study [10], we found that the graded association between the increasing number of risk factors within target range and lower risk of all-cause mortality in patients with T2D was stronger among younger individuals than older individuals. The greater reduction in mortality risk among younger individuals may be due to the age at which T2D was diagnosed [9]. Younger age at diagnosis of T2D is associated with higher mortality and cardiovascular risk [34], highlighting the need for, and the greater potential gains from, more aggressive interventions among younger patients with T2D, who generally had a lower number of risk factor within target range than older patients in this study.

Previous studies have reported the associations between adherence to a healthy lifestyle and lower mortality risk among patients with T2D [11, 33, 35]. Our study extended the previous evidence by including the evaluation of metabolic risk factor control simultaneously and demonstrating a progressive reduction in risk of all-cause and cause-specific mortality following the increasing number of lifestyle and metabolic risk factors within target range. We observed no significant interaction between the presence of T2D and risk factor targets achieved on mortality. Therefore, joint risk factor control is supposed to yield equivalent benefits in reducing the rate of mortality between individuals with and without T2D. In addition, among individuals with T2D the protective association of lifestyle risk factors on target with mortality was significant across all metabolic risk factor categories (data not shown). Together with existing evidence, our findings lend strong support to multifactorial risk factor intervention approaches in diabetes care and the implementation of lifestyle modification regardless of metabolic status.

Patients with T2D have significant losses of life expectancy than their counterparts without diabetes [4]. As far as we are aware, no previous study has shown how risk factor control influences life expectancy among patients with T2D compared with the general population. Our result that life expectancy in T2D gradually increased with an increasing number of risk factors within target range was align with the finding from a recent study showing an association between favourable cardiovascular health and improved life expectancy [36]. Of note, there was no significant difference in life expectancy at 50 years between individuals without T2D and patients with T2D who had 6–7 risk factors within target range. Our findings hold important clinical and public health implications suggesting that patients with T2D may have similar survival to the general population by achieving comprehensive risk factor targets across the life course.

Main strengths of this study included the large sample size and multiple data resources within the prospective UK Biobank cohort, which enabled us to perform detailed analyses on all-cause and cause-specific mortality in association with the combination of lifestyle and metabolic risk factors among patients with T2D. The robustness of the main results was confirmed in a range of sensitivity analyses. Our study also has some notable limitations. First, selected risk factors were evaluated at baseline visit, so their changes over time could not be captured. Nevertheless, the results remained stable after excluding undiagnosed patients with T2D for whom the lifestyle was assumed to change upon the awareness of diabetes. Second, since the assessment of lifestyle factors was mainly based on self-reported information, measurement errors and misclassification were inevitable, but such misclassification would likely biased findings toward the null. Third, participants excluded from the analyses due to missing data on risk factors were more likely to be less educated and more deprived. Given that the association between lifestyle and mortality risk is stronger with higher levels of socioeconomic deprivation [37], the effect estimates of risk factor target achievement in this study might have been underestimated. Moreover, when we repeated the analyses using multiple imputation for missing exposure and covariates, the results did not alter appreciably. Fourth, the nature of the observational study precludes causal inference and a direct comparison of the effects of treating risk factors, since we did not distinguish between patients with T2D who have achieved risk factors within target range by receiving treatment or not. Fifth, using the sum of the risk factors within target range assumes that all risk factors are equally relevant, which might ignore the varying magnitudes of associations between individual factors and mortality risk. In supplementary analysis, we generated a weighted risk factor score and observed similar results across weighted score categories. Sixth, lifestyle and metabolic risk factors might be influenced by severe diseases or poor health at baseline. Hence, we excluded participants with CVD or cancer from the analysis to reduce the possibility of reverse causation. Finally, participants in the UK Biobank were mostly White British. Further research is warranted to validate the generalisability of our findings to other ethnic groups.

## Conclusions

Our results showed that the risk of all-cause and cause-specific mortality among patients with T2D decreased stepwise with an increasing number of risk factors within target range. Patients with T2D who achieved 6 or more risk factors within target range had no significant excess risk of mortality or reduction in life expectancy compared with individuals without diabetes. These findings highlight the need for clinical practice and public health interventions targeting multiple risk factors to improve long-term survival among patients with T2D.

### Supplementary Information

**Additional file 1:** **Table S1.** Codes to identify prevalent diseases and cause-specific mortality. **Table S2.** Definition of risk factors with cutoffs. **Table S3.** Risk of mortality for participants with type 2 diabetes compared with those without diabetes. **Table S4.** Associations between individual risk factors and mortality risk among participants with type 2 diabetes. **Table S5.** Risk of mortality according to the number of risk factors within target range among participants with and without diabetes.** Table S6.** Risk of mortality per additional risk factor within target range by subgroups among participants with type 2 diabetes. **Table S7.** Estimated years of life gain at age 50 years by the number of risk factors within target range among participants with type 2 diabetes. **Table S8.** Results of sensitivity analyses with alternative definitions of risk factors or additional model adjustment. **Table S9.** Results of sensitivity analyses with people without diabetes who had different number of risk factors within target range as reference. **Table S10.** Results of sensitivity analyses according to the weighted score of risk factors within target range. **Table S11.** Results of sensitivity analyses for cause-specific mortality using competing risk regression. **Table S12.** Results of sensitivity analyses using multiple imputations to assign missing values of exposures and covariates. **Table S13.** Results of sensitivity analyses after excluding deaths within the first 2 years of follow-up. **Table S14.** Results of sensitivity analyses among participants with diagnosed diabetes. **Fig. S1.** Flow diagram of the study population. **Fig. S2.** Number of risk factors within target range among participants with and without diabetes. **Fig. S3.** Kaplan–Meier curves for cumulative rate of mortality according to the number of risk factors within target range among participants with and without diabetes.

## Data Availability

The data used in this current study are available from the UK Biobank data resources. Permissions are required in order to gain access to the UK Biobank data resources, subject to successful registration and application process. Further information can be found on the UK Biobank website (https://www.ukbiobank.ac.uk/).

## Abbreviations

CVD: Cardiovascular disease
HbA_1c_: Hemoglobin A_1c_
LDL: Low-density lipoprotein
T2D: Type 2 diabetes
TDI: Townsend deprivation index
