# Supplementary material for: Assessing the impact of type 2 diabetes on mortality and life expectancy according to the number of risk factor targets achieved: an observational study
Source: BMC Med. 2024 Mar 13;22:114. doi: 10.1186/s12916-024-03343-w (PMC10935790; doi:10.1186/s12916-024-03343-w)
Supplement: Supplementary file 1 — Additional file 1: Table S1. Codes to identify prevalent diseases and cause-specific mortality. Table S2. Definition of risk factors with cutoffs. Table S3. Risk of mortality for participants with type 2 diabetes compared with those without diabetes. Table S4. Associations between individual risk factors and mortality risk among participants with type 2 diabetes. Table S5. Risk of mortality according to the number of risk factors within target range among participants with and without diabetes. Table S6. Risk of mortality per additional risk factor within target range by subgroups among participants with type 2 diabetes. Table S7. Estimated years of life gain at age 50 years by the number of risk factors within target range among participants with type 2 diabetes. Table S8. Results of sensitivity analyses with alternative definitions of risk factors or additional model adjustment. Table S9. Results of sensitivity analyses with people without diabetes who had different number of risk factors within target range as reference. Table S10. Results of sensitivity analyses according to the weighted score of risk factors within target range. Table S11. Results of sensitivity analyses for cause-specific mortality using competing risk regression. Table S12. Results of sensitivity analyses using multiple imputations to assign missing values of exposures and covariates. Table S13. Results of sensitivity analyses after excluding deaths within the first 2 years of follow-up. Table S14. Results of sensitivity analyses among participants with diagnosed diabetes. Fig. S1. Flow diagram of the study population. Fig. S2. Number of risk factors within target range among participants with and without diabetes. Fig. S3. Kaplan–Meier curves for cumulative rate of mortality according to the number of risk factors within target range among participants with and without diabetes. [file 12916_2024_3343_MOESM1_ESM.docx]

**Additional file 1**

**Assessing the impact of type 2 diabetes on mortality and life expectancy according to the number of risk factor targets achieved: an observational study**

**Contents**

**Table S1**. Codes in the UK Biobank to identify prevalent diseases and cause-specific mortality.

**Table S2**. Definition of risk factors with cutoffs in the UK Biobank based on the guideline recommendations.

**Table S3**. Risk of mortality for participants with type 2 diabetes compared with those without diabetes.

**Table S4**. Associations between individual risk factors and mortality risk among participants with type 2 diabetes.

**Table S5**. Risk of mortality according to the number of risk factors within target range among participants with and without diabetes.

**Table S6**. Risk of mortality per additional risk factor within target range by subgroups among participants with type 2 diabetes.

**Table S7**. Estimated years of life gain at age 50 years by the number of risk factors within target range among participants with type 2 diabetes.

**Table S8**. Hazard ratios (95% CIs) of mortality risk according to the number of risk factors within target range among participants with type 2 diabetes compared to those without diabetes, with alternative definitions of risk factors or additional model adjustment.

**Table S9**. Hazard ratios (95% CIs) of mortality risk according to the number of risk factors within target range among participants with type 2 diabetes compared to those without diabetes who had different number of risk factors within target range as reference.

**Table S10**. Risk of mortality according to the weighted score of risk factors within target range among participants with type 2 diabetes compared to those without diabetes.

**Table S11**. Risk of cause-specific mortality according to the number of risk factors within target range among participants with type 2 diabetes compared with those without diabetes using competing risk regression.

**Table S12**. Risk of mortality according to the number of risk factors within target range among participants with type 2 diabetes compared to those without diabetes using multiple imputations with chained equations to assign missing values of exposures and covariates.

**Table S13**. Risk of mortality according to the number of risk factors within target range among participants with type 2 diabetes compared with those without diabetes after excluding deaths within the first 2 years of follow-up.

**Table S14**. Risk of mortality according to the number of risk factors within target range among participants with diagnosed diabetes compared with those without diabetes.

**Fig. S1**. Flow diagram of the study population.

**Fig. S2**. Number of risk factors within target range among participants with and without diabetes.

**Fig. S3**. Kaplan–Meier curves for cumulative rate of mortality according to the number of risk factors within target range among participants with and without diabetes.

**Table S1. Codes in the UK Biobank to identify prevalent diseases and cause-specific mortality**.

|  | ICD-9 | ICD-10 | Self-reported fields |
| --- | --- | --- | --- |
| Prevalent CVD* | 410-414, 430-434, 436 | I20-I25, I60-I64 | 6150 (1, 2, 3), 20002 (1074, 1075, 1081, 1583, 1086, 1491) |
| Prevalent cancer | 140-208 | C00-C97 | 20001 |
| CVD mortality |  | I00-I99 |  |
| Cancer mortality |  | C00-C97 |  |

*Includes coronary heart disease and stroke.

CVD, cardiovascular disease; ICD, International Classification of Diseases.

**Table S2. Definition of risk factors with cutoffs in the UK Biobank based on the guideline recommendations***.

| Risk factor | Source | On target | Not on target |
| --- | --- | --- | --- |
| Smoking | Touchscreen questionnaire at baseline | No current smoking | Current smoking |
| Physical activity† | Touchscreen questionnaire at baseline | ≥150 min/week moderate activity or ≥75 min/week vigorous activity or an equivalent combination | <150 min/week moderate and <75 min/week vigorous activity |
| Diet | Food frequency questionnaire at baseline | At least 4 of the following 7 food groups:  1. Fruits: ≥ 3 servings/day  2. Vegetables: ≥ 3 servings/day  3. (Shell)fish: ≥ 2 servings/week 4. Processed meats: ≤1 servings/week 5. Unprocessed meats: ≤2 serving/week  6. Whole grains: ≥3 servings/day  7. Refined grains: ≤2 servings/day | <4 of the 7 recommended food groups |
| BMI | Physical measures | ≥20 and <25 kg/m^2^ | <20 or ≥25 kg/m^2^ |
| Blood pressure‡ | Physical measures | Systolic <140 mmHg and diastolic <90 mmHg | Systolic ≥140 mmHg or diastolic ≥90 mmHg |
| Glycated hemoglobin | Blood biochemistry | <53 mmol/mol (<7%) | ≥53 mmol/mol (≥7%) |
| Total cholesterol | Blood biochemistry | <5.2 mmol/L (<200 mg/dL) | ≥5.2 mmol/L (≥200 mg/dL) |

*Seven risk factors were defined as being within target range from the recommendations in current clinical guidelines (1-3). Detailed information on questionnaires, physical measurements, and biochemistry assays can be obtained through the UK Biobank website (https://biobank.ndph.ox.ac.uk/showcase/).

†Physical activity was assessed using adapted questions from the validated short International Physical Activity Questionnaire (IPAQ). ‡Blood pressure was measured twice in the UK Biobank, and the average of the readings was used in the analyses.

**References**

1. Lloyd-Jones DM, Hong Y, Labarthe D, et al; American Heart Association Strategic Planning Task Force and Statistics Committee. Defining and setting national goals for cardiovascular health promotion and disease reduction: the American Heart Association’s strategic Impact Goal through 2020 and beyond. Circulation. 2010;121(4):586-613.

2. Mozaffarian D. Dietary and policy priorities for cardiovascular disease, diabetes, and obesity: a comprehensive review. Circulation. 2016;133(2): 187-225.

3. The National Institute for Health and Care Excellence (NICE). Type 2 diabetes in adults: management - NICE guideline NG28. 2017. Accessed May 8, 2023. https://www.nice.org.uk/guidance/ng28.

**Table S3. Risk of mortality for participants with type 2 diabetes compared with those without diabetes**.

|  | Deaths/N | Deaths per 1000 person-years | Crude HR (95% CI) | Adjusted HR* (95% CI) |
| --- | --- | --- | --- | --- |
| **All-cause mortality** |  |  |  |  |
| Without diabetes | 18 505/302 833 | 4.5 | 1 (reference) | 1 (reference) |
| Type 2 diabetes | 2105/14 162 | 11.4 | 1.97 (1.89-2.07) | 1.60 (1.53-1.68) |
| **CVD mortality** |  |  |  |  |
| Without diabetes | 3389/302 833 | 0.8 | 1 (reference) | 1 (reference) |
| Type 2 diabetes | 513/14 162 | 2.8 | 2.61 (2.37-2.86) | 1.78 (1.61-1.96) |
| **Cancer mortality** |  |  |  |  |
| Without diabetes | 9396/302 833 | 2.3 | 1 (reference) | 1 (reference) |
| Type 2 diabetes | 809/14 162 | 4.4 | 1.51 (1.41-1.63) | 1.32 (1.22-1.42) |

*Cox proportional hazards regression with age as time scale was adjusted for sex, ethnicity, education, Townsend deprivation index, smoking, physical activity, diet, and BMI.

CVD, cardiovascular disease; HR, hazard ratio.

**Table S4. Associations between individual risk factors and mortality risk among participants with type 2 diabetes**.

|  |  | **All-cause mortality** | | **CVD mortality** | | **Cancer mortality** | |
| --- | --- | --- | --- | --- | --- | --- | --- |
|  | **N** | **Deaths** | **HR (95% CI)*** | **Deaths** | **HR (95% CI)** | **Deaths** | **HR (95% CI)** |
| **Smoking status** |  |  |  |  |  |  |  |
| Not on target | 1509 | 327 | 1 (reference) | 90 | 1 (reference) | 139 | 1 (reference) |
| On target | 12 653 | 1778 | 0.58 (0.51-0.66) | 423 | 0.53 (0.42-0.67) | 670 | 0.50 (0.41-0.60) |
| **Physical activity** |  |  |  |  |  |  |  |
| Not on target | 7615 | 1200 | 1 (reference) | 310 | 1 (reference) | 443 | 1 (reference) |
| On target | 6547 | 902 | 0.82 (0.75-0.89) | 203 | 0.72 (0.60-0.86) | 366 | 0.90 (0.78-1.03) |
| **Diet** |  |  |  |  |  |  |  |
| Not on target | 8591 | 1323 | 1 (reference) | 345 | 1 (reference) | 493 | 1 (reference) |
| On target | 5571 | 782 | 0.95 (0.87-1.04) | 168 | 0.82 (0.68-0.99) | 316 | 1.03 (0.89-1.18) |
| **BMI** |  |  |  |  |  |  |  |
| Not on target | 12 649 | 1922 | 1 (reference) | 475 | 1 (reference) | 740 | 1 (reference) |
| On target | 1513 | 183 | 0.81 (0.69-0.94) | 38 | 0.69 (0.49-0.96) | 69 | 0.79 (0.62-1.02) |
| **Blood pressure** |  |  |  |  |  |  |  |
| Not on target | 8039 | 1320 | 1 (reference) | 339 | 1 (reference) | 512 | 1 (reference) |
| On target | 6123 | 785 | 0.93 (0.85-1.02) | 174 | 0.80 (0.67-0.97) | 297 | 0.90 (0.78-1.04) |
| **HbA_1c_** |  |  |  |  |  |  |  |
| Not on target | 5238 | 831 | 1 (reference) | 205 | 1 (reference) | 306 | 1 (reference) |
| On target | 8924 | 1274 | 0.88 (0.80-0.96) | 308 | 0.90 (0.75-1.08) | 503 | 0.93 (0.80-1.07) |
| **Total cholesterol** |  |  |  |  |  |  |  |
| Not on target | 4472 | 571 | 1 (reference) | 144 | 1 (reference) | 221 | 1 (reference) |
| On target | 9690 | 1534 | 1.03 (0.93-1.14) | 369 | 0.97 (0.79-1.18) | 588 | 1.07 (0.91-1.25) |

*Hazard ratio was estimated using Cox proportional hazards regression with age as time scale and adjusted for sex, ethnicity, education, Townsend deprivation index, and diabetes duration. Individual risk factors were mutually adjusted.

CVD, cardiovascular disease; HR, hazard ratio.

**Table S5. Risk of mortality according to the number of risk factors within target range among participants with and without diabetes**.

|  | **No. of risk factors within target range** | | | | | | |
| --- | --- | --- | --- | --- | --- | --- | --- |
|  | **0-1** | **2** | **3** | **4** | **5** | **6-7** | **Per 1-number increase** |
| **With diabetes** |  |  |  |  |  |  |  |
| **All-cause mortality** |  |  |  |  |  |  |  |
| Deaths/N | 114/594 | 355/2106 | 632/3947 | 582/4122 | 337/2507 | 85/886 |  |
| HR (95% CI) | 1 (reference) | 0.80 (0.64-0.98) | 0.70 (0.57-0.85) | 0.61 (0.50-0.75) | 0.58 (0.47-0.72) | 0.43 (0.32-0.57) | 0.88 (0.85-0.91) |
| **CVD mortality** |  |  |  |  |  |  |  |
| Deaths/N | 35/594 | 92/2106 | 174/3947 | 136/4122 | 57/2507 | 19/886 |  |
| HR (95% CI) | 1 (reference) | 0.69 (0.47-1.01) | 0.64 (0.45-0.93) | 0.48 (0.33-0.70) | 0.33 (0.22-0.51) | 0.32 (0.18-0.57) | 0.79 (0.74-0.85) |
| **Cancer mortality** |  |  |  |  |  |  |  |
| Deaths/N | 41/594 | 133/2106 | 241/3947 | 226/4122 | 132/2507 | 36/886 |  |
| HR (95% CI) | 1 (reference) | 0.84 (0.59-1.19) | 0.75 (0.54-1.04) | 0.67 (0.48-0.93) | 0.64 (0.45-0.92) | 0.51 (0.33-0.81) | 0.90 (0.85-0.95) |
| **Without diabetes** |  |  |  |  |  |  |  |
| **All-cause mortality** |  |  |  |  |  |  |  |
| Deaths/N | 338/2491 | 2496/27 777 | 5189/72 552 | 5633/92 705 | 3458/69 835 | 1391/37 473 |  |
| HR (95% CI) | 1 (reference) | 0.61 (0.55-0.68) | 0.49 (0.44-0.55) | 0.43 (0.39-0.48) | 0.39 (0.35-0.43) | 0.35 (0.31-0.39) | 0.86 (0.85-0.88) |
| **CVD mortality** |  |  |  |  |  |  |  |
| Deaths/N | 94/2491 | 515/27 777 | 1020/72 552 | 1026/92 705 | 550/69 835 | 184/37 473 |  |
| HR (95% CI) | 1 (reference) | 0.46 (0.37-0.58) | 0.36 (0.29-0.44) | 0.30 (0.24-0.37) | 0.24 (0.19-0.30) | 0.18 (0.14-0.23) | 0.78 (0.76-0.81) |
| **Cancer mortality** |  |  |  |  |  |  |  |
| Deaths/N | 151/2491 | 1252/27 777 | 2571/72 552 | 2811/92 705 | 1839/69 835 | 772/37 473 |  |
| HR (95% CI) | 1 (reference) | 0.68 (0.57-0.80) | 0.53 (0.45-0.63) | 0.47 (0.40-0.56) | 0.44 (0.38-0.52) | 0.41 (0.34-0.48) | 0.88 (0.87-0.90) |

*Hazard ratio was estimated using Cox proportional hazards regression with age as time scale and adjusted for sex, ethnicity, education, Townsend deprivation index, and diabetes duration (for participants with diabetes only). *P* value for interaction between diabetes status and per additional risk factor within target range was 0.58 for all-cause mortality, 0.91 for CVD mortality, and 0.67 for cancer mortality.

CVD, cardiovascular disease; HR, hazard ratio.

**Table S6. Risk of mortality per additional risk factor within target range by subgroups among participants with type 2 diabetes**.

|  | **N** | **All-cause mortality** | | | **CVD mortality** | | | **Cancer mortality** | | |
| --- | --- | --- | --- | --- | --- | --- | --- | --- | --- | --- |
|  |  | **Deaths** | **HR (95% CI)*** | ***P* for**  **interaction** | **Deaths** | **HR (95% CI)** | ***P* for**  **interaction** | **Deaths** | **HR (95% CI)** | ***P* for**  **interaction** |
| **Age, years** |  |  |  |  |  |  |  |  |  |  |
| <60 | 6326 | 502 | 0.83 (0.77-0.89) | 0.046 | 136 | 0.77 (0.67-0.88) | 0.48 | 205 | 0.82 (0.74-0.92) | 0.10 |
| ≥60 | 7836 | 1603 | 0.89 (0.86-0.93) |  | 377 | 0.80 (0.74-0.87) |  | 604 | 0.93 (0.87-0.99) |  |
| **Sex** |  |  |  |  |  |  |  |  |  |  |
| Female | 5585 | 605 | 0.90 (0.84-0.96) | 0.23 | 121 | 0.83 (0.72-0.96) | 0.42 | 251 | 0.92 (0.83-1.02) | 0.43 |
| Male | 8577 | 1500 | 0.87 (0.83-0.90) |  | 392 | 0.78 (0.72-0.85) |  | 558 | 0.89 (0.83-0.95) |  |
| **Education** |  |  |  |  |  |  |  |  |  |  |
| College or university degree | 3711 | 450 | 0.87 (0.81-0.93) | 0.94 | 113 | 0.74 (0.64-0.86) | 0.52 | 178 | 0.87 (0.77-0.98) | 0.61 |
| Other | 10284 | 1624 | 0.88 (0.84-0.92) |  | 389 | 0.80 (0.74-0.87) |  | 622 | 0.91 (0.85-0.97) |  |
| **Townsend deprivation index** |  |  |  |  |  |  |  |  |  |  |
| <Median | 7081 | 979 | 0.91 (0.87-0.96) | 0.028 | 231 | 0.83 (0.75-0.92) | 0.18 | 402 | 0.96 (0.89-1.04) | 0.017 |
| ≥Median | 7081 | 1126 | 0.84 (0.80-0.89) |  | 282 | 0.76 (0.69-0.84) |  | 407 | 0.84 (0.77-0.91) |  |
| **Diabetes duration, years** |  |  |  |  |  |  |  |  |  |  |
| 0 to <1 | 4213 | 517 | 0.86 (0.80-0.92) | 0.36 | 125 | 0.80 (0.69-0.92) | 0.59 | 218 | 0.84 (0.76-0.94) | 0.12 |
| 1 to <5 | 5231 | 742 | 0.87 (0.82-0.93) |  | 171 | 0.76 (0.66-0.86) |  | 280 | 0.91 (0.82-1.00) |  |
| ≥5 | 4718 | 846 | 0.88 (0.83-0.94) |  | 217 | 0.81 (0.72-0.91) |  | 311 | 0.94 (0.85-1.03) |  |
| **Diabetes medication use†** |  |  |  |  |  |  |  |  |  |  |
| Yes | 7869 | 1249 | 0.87 (0.83-0.91) | 0.73 | 322 | 0.79 (0.72-0.86) | 0.90 | 440 | 0.92 (0.85-1.00) | 0.42 |
| No | 6293 | 856 | 0.88 (0.83-0.93) |  | 191 | 0.79 (0.70-0.88) |  | 369 | 0.88 (0.81-0.95) |  |

*Hazard ratio was estimated using Cox proportional hazards regression with age as time scale and adjusted for sex, ethnicity, education, Townsend deprivation index, and diabetes duration. The strata variable was not included in the model when stratifying by itself.

†Diabetes medication included oral antidiabetic drug or insulin use.

CVD, cardiovascular disease; HR, hazard ratio.

**Table S7. Estimated years of life gain at age 50 years by the number of risk factors within target range among participants with type 2 diabetes**.

|  | **Years of life gain**  **(95% CI)*** | **Years of life gain between adjacent groups (95% CI)** | **Mean years of life gain per additional risk factor on target** |
| --- | --- | --- | --- |
| **Number of risk factors on target** |  |  |  |
| 0-1 | 0 (reference) |  | 1.30 (0.32-2.28) |
| 2 | 1.95 (0.22-3.69) | 1.95 (0.22 to 3.69) |  |
| 3 | 3.03 (1.33-4.74) | 1.08 (0.02 to 2.14) |  |
| 4 | 4.15 (2.35-5.94) | 1.12 (0.19 to 2.04) |  |
| 5 | 4.50 (2.59-6.41) | 0.35 (-0.72 to 1.42) |  |
| 6 | 6.79 (4.23-9.34) | 2.29 (0.35 to 4.22) |  |
| 7 | 7.79 (1.89-13.7) | 1.00 (-4.78 to 6.78) |  |

*Years of life lost was calculated using flexible parametric survival models with age as time scale and adjusted for sex, ethnicity, education, Townsend deprivation index, and diabetes duration.

**Table S8. Hazard ratios (95% CIs) of mortality risk according to the number of risk factors within target range among participants with type 2 diabetes compared to those without diabetes, with alternative definitions of risk factors or additional model adjustment***.

|  | **All-cause mortality** | **CVD mortality** | **Cancer mortality** |
| --- | --- | --- | --- |
| **Cutoff value for HbA_1c_, <48 mmol/mol (<6.5%)** | | | |
| Without diabetes | 1 (reference) | 1 (reference) | 1 (reference) |
| 0-1 risk factor on target | 2.46 (2.11-2.86) | 3.42 (2.56-4.58) | 2.01 (1.57-2.56) |
| 2 risk factors on target | 1.95 (1.77-2.15) | 2.75 (2.28-3.30) | 1.53 (1.30-1.79) |
| 3 risk factors on target | 1.84 (1.70-1.99) | 2.41 (2.06-2.82) | 1.44 (1.26-1.64) |
| 4 risk factors on target | 1.65 (1.51-1.80) | 1.94 (1.62-2.31) | 1.37 (1.19-1.57) |
| 5 risk factors on target | 1.55 (1.38-1.75) | 1.39 (1.04-1.85) | 1.23 (1.01-1.49) |
| 6-7 risk factors on target | 1.04 (0.81-1.35) | 0.90 (0.48-1.67) | 0.99 (0.68-1.45) |
| **Cutoff value for blood pressure, <130/80 mmHg** | | | |
| Without diabetes | 1 (reference) | 1 (reference) | 1 (reference) |
| 0-1 risk factor on target | 2.59 (2.20-3.06) | 3.80 (2.79-5.17) | 1.93 (1.47-2.54) |
| 2 risk factors on target | 2.00 (1.81-2.20) | 2.79 (2.32-3.36) | 1.53 (1.31-1.79) |
| 3 risk factors on target | 1.86 (1.72-2.00) | 2.40 (2.06-2.80) | 1.53 (1.36-1.73) |
| 4 risk factors on target | 1.59 (1.46-1.74) | 1.88 (1.57-2.24) | 1.25 (1.09-1.44) |
| 5 risk factors on target | 1.44 (1.27-1.63) | 1.10 (0.79-1.51) | 1.24 (1.03-1.52) |
| 6-7 risk factors on target | 1.23 (0.92-1.65) | 1.65 (0.94-2.92) | 1.05 (0.67-1.65) |
| **Cutoff value for BMI, ≥20 and <30 kg/m^2^** | | | |
| Without diabetes | 1 (reference) | 1 (reference) | 1 (reference) |
| 0-1 risk factor on target | 2.98 (2.40-3.71) | 4.94 (3.36-7.27) | 2.19 (1.52-3.15) |
| 2 risk factors on target | 2.47 (2.20-2.77) | 3.41 (2.72-4.27) | 1.81 (1.49-2.19) |
| 3 risk factors on target | 1.85 (1.69-2.02) | 2.73 (2.32-3.22) | 1.48 (1.28-1.70) |
| 4 risk factors on target | 1.81 (1.67-1.96) | 2.06 (1.74-2.45) | 1.48 (1.30-1.68) |
| 5 risk factors on target | 1.44 (1.30-1.59) | 1.47 (1.17-1.83) | 1.14 (0.97-1.34) |
| 6-7 risk factors on target | 1.32 (1.15-1.52) | 1.28 (0.94-1.75) | 1.21 (0.98-1.49) |
| **Excluding smoking as a risk factor** | | | |
| Without diabetes | 1 (reference) | 1 (reference) | 1 (reference) |
| 0-1 risk factor on target | 2.11 (1.90-2.34) | 2.73 (2.22-3.35) | 1.54 (1.30-1.83) |
| 2 risk factors on target | 1.89 (1.74-2.05) | 2.72 (2.34-3.17) | 1.56 (1.37-1.77) |
| 3 risk factors on target | 1.69 (1.56-1.83) | 2.10 (1.79-2.47) | 1.31 (1.15-1.49) |
| 4 risk factors on target | 1.60 (1.44-1.77) | 1.37 (1.07-1.76) | 1.38 (1.18-1.62) |
| 5-6 risk factors on target | 1.25 (1.03-1.52) | 1.34 (0.87-2.06) | 1.12 (0.83-1.51) |
| **Excluding physical activity as a risk factor** | | | |
| Without diabetes | 1 (reference) | 1 (reference) | 1 (reference) |
| 0-1 risk factor on target | 2.23 (1.91-2.60) | 3.46 (2.62-4.58) | 1.69 (1.31-2.18) |
| 2 risk factors on target | 2.07 (1.90-2.26) | 2.63 (2.21-3.13) | 1.66 (1.44-1.91) |
| 3 risk factors on target | 1.70 (1.58-1.83) | 2.21 (1.91-2.57) | 1.39 (1.23-1.56) |
| 4 risk factors on target | 1.65 (1.51-1.80) | 1.80 (1.49-2.17) | 1.34 (1.17-1.54) |
| 5-6 risk factors on target | 1.36 (1.17-1.58) | 1.34 (0.95-1.88) | 1.05 (0.83-1.34) |
| **Excluding diet as a risk factor** | | | |
| Without diabetes | 1 (reference) | 1 (reference) | 1 (reference) |
| 0-1 risk factor on target | 2.72 (2.33-3.17) | 4.03 (3.02-5.38) | 2.04 (1.58-2.63) |
| 2 risk factors on target | 2.01 (1.83-2.21) | 2.67 (2.22-3.21) | 1.62 (1.39-1.88) |
| 3 risk factors on target | 1.80 (1.67-1.93) | 2.35 (2.03-2.72) | 1.46 (1.29-1.64) |
| 4 risk factors on target | 1.56 (1.43-1.70) | 1.70 (1.41-2.04) | 1.22 (1.06-1.40) |
| 5-6 risk factors on target | 1.35 (1.17-1.55) | 1.27 (0.91-1.77) | 1.17 (0.94-1.46) |
| **Excluding BMI as a risk factor** | | | |
| Without diabetes | 1 (reference) | 1 (reference) | 1 (reference) |
| 0-1 risk factor on target | 2.71 (2.27-3.24) | 4.36 (3.18-5.98) | 1.98 (1.47-2.67) |
| 2 risk factors on target | 2.14 (1.93-2.37) | 2.79 (2.28-3.41) | 1.65 (1.39-1.95) |
| 3 risk factors on target | 1.80 (1.66-1.95) | 2.45 (2.10-2.86) | 1.49 (1.31-1.68) |
| 4 risk factors on target | 1.63 (1.50-1.77) | 1.83 (1.54-2.18) | 1.31 (1.15-1.49) |
| 5-6 risk factors on target | 1.46 (1.31-1.62) | 1.44 (1.13-1.82) | 1.20 (1.02-1.42) |
| **Excluding HbA_1c_ as a risk factor** | | | |
| Without diabetes | 1 (reference) | 1 (reference) | 1 (reference) |
| 0-1 risk factor on target | 2.39 (2.10-2.73) | 3.70 (2.90-4.71) | 1.96 (1.59-2.42) |
| 2 risk factors on target | 1.93 (1.78-2.10) | 2.70 (2.30-3.16) | 1.51 (1.31-1.72) |
| 3 risk factors on target | 1.73 (1.60-1.86) | 2.04 (1.74-2.38) | 1.38 (1.22-1.56) |
| 4 risk factors on target | 1.58 (1.44-1.73) | 1.60 (1.29-1.97) | 1.29 (1.12-1.50) |
| 5-6 risk factors on target | 1.31 (1.10-1.56) | 1.37 (0.93-2.01) | 1.11 (0.85-1.46) |
| **Excluding blood pressure as a risk factor** | | | |
| Without diabetes | 1 (reference) | 1 (reference) | 1 (reference) |
| 0-1 risk factor on target | 2.58 (2.21-3.02) | 3.53 (2.61-4.77) | 2.03 (1.58-2.61) |
| 2 risk factors on target | 2.06 (1.88-2.26) | 2.78 (2.33-3.32) | 1.54 (1.32-1.79) |
| 3 risk factors on target | 1.81 (1.68-1.95) | 2.46 (2.13-2.85) | 1.48 (1.31-1.66) |
| 4 risk factors on target | 1.55 (1.42-1.69) | 1.62 (1.34-1.96) | 1.24 (1.07-1.42) |
| 5-6 risk factors on target | 1.31 (1.14-1.51) | 1.20 (0.87-1.66) | 1.21 (0.98-1.49) |
| **Excluding total cholesterol as a risk factor** | | | |
| Without diabetes | 1 (reference) | 1 (reference) | 1 (reference) |
| 0-1 risk factor on target | 2.59 (2.31-2.90) | 3.71 (3.00-4.58) | 1.98 (1.64-2.38) |
| 2 risk factors on target | 1.92 (1.77-2.08) | 2.58 (2.20-3.02) | 1.52 (1.33-1.73) |
| 3 risk factors on target | 1.67 (1.55-1.81) | 2.12 (1.81-2.48) | 1.34 (1.18-1.51) |
| 4 risk factors on target | 1.56 (1.42-1.72) | 1.37 (1.08-1.74) | 1.34 (1.15-1.56) |
| 5-6 risk factors on target | 1.15 (0.95-1.39) | 1.33 (0.89-1.98) | 0.92 (0.68-1.24) |
| **Sleep duration as additional risk factor, cutoff value 7-8 h/day** | | | |
| Without diabetes | 1 (reference) | 1 (reference) | 1 (reference) |
| 0-2 risk factors on target | 2.65 (2.35-2.98) | 3.65 (2.90-4.59) | 2.09 (1.72-2.53) |
| 3 risk factors on target | 2.03 (1.85-2.23) | 2.95 (2.48-3.51) | 1.54 (1.33-1.80) |
| 4 risk factors on target | 1.78 (1.64-1.93) | 2.28 (1.94-2.68) | 1.44 (1.26-1.64) |
| 5 risk factors on target | 1.46 (1.33-1.60) | 1.49 (1.21-1.84) | 1.18 (1.02-1.38) |
| 6 risk factors on target | 1.50 (1.33-1.71) | 1.43 (1.07-1.92) | 1.27 (1.04-1.55) |
| 7-8 risk factors on target | 1.19 (0.92-1.54) | 1.16 (0.64-2.10) | 1.18 (0.82-1.71) |
| **Additional adjustment for estimated glomerular filtration rate** | | | |
| Without diabetes | 1 (reference) | 1 (reference) | 1 (reference) |
| 0-1 risk factor on target | 2.69 (2.23-3.23) | 4.28 (3.07-5.98) | 1.98 (1.46-2.70) |
| 2 risk factors on target | 2.13 (1.92-2.37) | 2.86 (2.32-3.52) | 1.66 (1.39-1.97) |
| 3 risk factors on target | 1.86 (1.72-2.01) | 2.58 (2.22-3.01) | 1.48 (1.30-1.69) |
| 4 risk factors on target | 1.63 (1.50-1.77) | 1.93 (1.62-2.29) | 1.32 (1.16-1.51) |
| 5 risk factors on target | 1.56 (1.40-1.74) | 1.35 (1.04-1.75) | 1.27 (1.07-1.51) |
| 6-7 risk factors on target | 1.15 (0.93-1.43) | 1.33 (0.85-2.10) | 1.01 (0.73-1.40) |
| **Additional adjustment for prevalent morbidity†** | | | |
| Without diabetes | 1 (reference) | 1 (reference) | 1 (reference) |
| 0-1 risk factor on target | 2.69 (2.24-3.23) | 4.17 (2.98-5.82) | 2.01 (1.48-2.73) |
| 2 risk factors on target | 2.11 (1.90-2.35) | 2.79 (2.26-3.43) | 1.66 (1.40-1.97) |
| 3 risk factors on target | 1.84 (1.70-2.00) | 2.56 (2.20-2.99) | 1.49 (1.31-1.69) |
| 4 risk factors on target | 1.62 (1.49-1.76) | 1.92 (1.61-2.28) | 1.32 (1.16-1.51) |
| 5 risk factors on target | 1.55 (1.39-1.73) | 1.34 (1.03-1.74) | 1.27 (1.07-1.51) |
| 6-7 risk factors on target | 1.16 (0.94-1.43) | 1.32 (0.84-2.08) | 1.02 (0.73-1.41) |

Seven risk factors on target were no current smoking, physical activity at goal, healthy diet, BMI ≥20 and <25 kg/m², blood pressure <140/90 mmHg, HbA_1c_ <53 mmol/mol (<7%), and total cholesterol <5.2 mmol/L (<200 mg/dL).

*Hazard ratio was estimated using Cox proportional hazards regression with age as time scale and adjusted for sex, ethnicity, education, and Townsend deprivation index.

†Prevalent morbidity included depression, schizophrenia, cognitive impartment, dementia, Parkinson’s disease, chronic obstructive pulmonary disease, chronic asthma, chronic liver diseases, inflammatory bowel diseases, and arthritis that was ascertained during a nurse-led interview at baseline.

CVD, cardiovascular disease.

**Table S9. Hazard ratios (95% CIs) of mortality risk according to the number of risk factors within target range among participants with type 2 diabetes compared to those without diabetes who had different number of risk factors within target range as reference*.**

|  | **All-cause mortality** | **CVD mortality** | **Cancer mortality** |
| --- | --- | --- | --- |
| **Individuals without diabetes who had 0-3 risk factors within target range as reference** | | | |
| 0-1 risk factor on target | 2.28 (1.89-2.74) | 3.25 (2.32-4.55) | 1.71 (1.26-2.33) |
| 2 risk factors on target | 1.82 (1.63-2.02) | 2.21 (1.79-2.74) | 1.43 (1.20-1.70) |
| 3 risk factors on target | 1.59 (1.46-1.72) | 2.05 (1.75-2.41) | 1.27 (1.12-1.45) |
| 4 risk factors on target | 1.39 (1.27-1.51) | 1.53 (1.28-1.83) | 1.13 (0.99-1.29) |
| 5 risk factors on target | 1.33 (1.19-1.49) | 1.07 (0.82-1.40) | 1.09 (0.92-1.30) |
| 6-7 risk factors on target | 0.99 (0.80-1.22) | 1.04 (0.66-1.64) | 0.87 (0.63-1.21) |
| **Individuals without diabetes who had 4 risk factors within target range as reference**† | | | |
| 0-1 risk factor on target | 2.86 (2.38-3.45) | 4.52 (3.22-6.34) | 2.16 (1.59-2.94) |
| 2 risk factors on target | 2.26 (2.03-2.51) | 3.03 (2.44-3.75) | 1.79 (1.50-2.13) |
| 3 risk factors on target | 1.97 (1.81-2.14) | 2.79 (2.37-3.29) | 1.60 (1.41-1.83) |
| 4 risk factors on target | 1.72 (1.58-1.87) | 2.07 (1.73-2.49) | 1.42 (1.24-1.63) |
| 5 risk factors on target | 1.64 (1.47-1.83) | 1.44 (1.10-1.89) | 1.37 (1.15-1.63) |
| 6-7 risk factors on target | 1.21 (0.98-1.51) | 1.41 (0.89-2.22) | 1.09 (0.79-1.52) |
| **Individuals without diabetes who had 5-7 risk factors within target range as reference** | | | |
| 0-1 risk factor on target | 3.35 (2.78-4.04) | 6.06 (4.31-8.53) | 2.42 (1.77-3.29) |
| 2 risk factors on target | 2.63 (2.36-2.93) | 4.04 (3.24-5.03) | 1.98 (1.66-2.36) |
| 3 risk factors on target | 2.29 (2.10-2.49) | 3.72 (3.14-4.40) | 1.78 (1.55-2.03) |
| 4 risk factors on target | 1.99 (1.83-2.18) | 2.75 (2.28-3.32) | 1.57 (1.37-1.81) |
| 5 risk factors on target | 1.90 (1.70-2.12) | 1.92 (1.46-2.52) | 1.51 (1.26-1.80) |
| 6-7 risk factors on target | 1.40 (1.13-1.74) | 1.86 (1.18-2.94) | 1.20 (0.86-1.67) |

*Hazard ratio was estimated using Cox proportional hazards regression with age as time scale and adjusted for sex, ethnicity, education, and Townsend deprivation index.

†The median number of risk factors within target range among individuals without diabetes.

**Table S10.** **Risk of mortality according to the weighted score of risk factors within target range among participants with type 2 diabetes compared to those without diabetes**.

|  | **Individuals**  **without diabetes** | **Weighted score category of risk factors within target range among individuals with diabetes*** | | | | | |
| --- | --- | --- | --- | --- | --- | --- | --- |
|  |  | **1** | **2** | **3** | **4** | **5** | **6** |
| **All-cause mortality** |  |  |  |  |  |  |  |
| Deaths/N | 18 505/302 833 | 148/637 | 397/2129 | 565/3804 | 620/4282 | 260/2149 | 115/1161 |
| Deaths per 1000 person-years | 4.5 | 18.3 | 14.4 | 11.4 | 11.0 | 9.2 | 7.5 |
| HR (95% CI)† | 1 (reference) | 3.18 (2.70-3.74) | 2.36 (2.13-2.60) | 1.76 (1.62-1.91) | 1.65 (1.52-1.79) | 1.40 (1.24-1.59) | 1.18 (0.98-1.42) |
| **CVD mortality** |  |  |  |  |  |  |  |
| Deaths/N | 3389/302 833 | 45/637 | 101/2129 | 145/3804 | 151/4282 | 51/2149 | 20/1161 |
| Deaths per 1000 person-years | 0.8 | 5.6 | 3.7 | 2.9 | 2.7 | 1.8 | 1.3 |
| HR (95% CI) | 1 (reference) | 4.76 (3.54-6.39) | 3.00 (2.46-3.66) | 2.31 (1.95-2.72) | 2.03 (1.72-2.39) | 1.42 (1.07-1.87) | 1.05 (0.67-1.63) |
| **Cancer mortality** |  |  |  |  |  |  |  |
| Deaths/N | 9396/302 833 | 62/637 | 147/2129 | 224/3804 | 222/4282 | 105/2149 | 49/1161 |
| Deaths per 1000 person-years | 2.3 | 7.7 | 5.3 | 4.5 | 3.9 | 3.7 | 3.2 |
| HR (95% CI) | 1 (reference) | 2.82 (2.20-3.62) | 1.83 (1.55-2.15) | 1.45 (1.27-1.66) | 1.24 (1.09-1.42) | 1.18 (0.97-1.43) | 1.05 (0.79-1.38) |

*The weighted score was calculated as follows: weighted score = (β1× factor1 + β2 × factor2 +…+ β7× factor7) × (7/sum of the β coefficients). β coefficients of each risk factor were estimated using Cox proportional hazards model that included all seven risk factors and all-cause mortality as an outcome. The weighted score was grouped into six ordered categories (1-6) based on the distribution of the number of risk factors within target range in the main analysis. A higher level indicates a higher number of risk factors within target range.

†Hazard ratio was estimated using Cox proportional hazards regression with age as time scale and adjusted for sex, ethnicity, education, and Townsend deprivation index.

CVD, cardiovascular disease; HR, hazard ratio.

**Table S11. Risk of cause-specific mortality according to the number of risk factors within target range among participants with type 2 diabetes compared with those without diabetes using competing risk regression**.

|  | **Individuals**  **without diabetes** | **No. of risk factors within target range among individuals with diabetes** | | | | | |
| --- | --- | --- | --- | --- | --- | --- | --- |
|  |  | **0-1** | **2** | **3** | **4** | **5** | **6-7** |
| **CVD mortality** |  |  |  |  |  |  |  |
| Deaths/N | 3389/302 833 | 35/594 | 92/2106 | 174/3947 | 136/4122 | 57/2507 | 19/886 |
| Deaths per 1000 person-years | 0.8 | 4.6 | 3.3 | 3.4 | 2.5 | 1.7 | 1.6 |
| HR (95% CI)* | 1 (reference) | 3.95 (2.83-5.52) | 2.66 (2.16-3.29) | 2.49 (2.13-2.91) | 1.87 (1.58-2.23) | 1.30 (1.00-1.70) | 1.31 (0.83-2.06) |
| **Cancer mortality** |  |  |  |  |  |  |  |
| Deaths/N | 9396/302 833 | 41/594 | 133/2106 | 241/3947 | 226/4122 | 132/2507 | 36/886 |
| Deaths per 1000 person-years | 2.3 | 5.3 | 4.8 | 4.7 | 4.2 | 4.0 | 3.1 |
| HR (95% CI) | 1 (reference) | 1.89 (1.39-2.58) | 1.60 (1.35-1.90) | 1.44 (1.27-1.64) | 1.29 (1.13-1.48) | 1.24 (1.05-1.48) | 1.01 (0.73-1.40) |

*Subdistribution hazard ratios were calculated using Fine and Gray proportional subdistribution hazards regression models with age as time scale and adjusted for sex, ethnicity, education, and Townsend deprivation index.

CVD, cardiovascular disease; HR, hazard ratio.

**Table S12. Risk of mortality according to the number of risk factors within target range among participants with type 2 diabetes compared to those without diabetes using multiple imputations with chained equations to assign missing values of exposures and covariates**.

|  | **Individuals**  **without diabetes** | **No. of risk factors within target range among individuals with diabetes** | | | | | |
| --- | --- | --- | --- | --- | --- | --- | --- |
|  |  | **0-1** | **2** | **3** | **4** | **5** | **6-7** |
| **All-cause mortality** |  |  |  |  |  |  |  |
| Deaths/N | 26 456/406 076 | 210/1029 | 593/3313 | 959/5795 | 842/5709 | 442/3251 | 114/1137 |
| Deaths per 1000 person-years | 4.8 | 15.8 | 13.7 | 12.7 | 11.2 | 10.4 | 7.5 |
| HR (95% CI)* | 1 (reference) | 2.52 (2.17-2.91) | 2.08 (1.91-2.27) | 1.82 (1.70-1.94) | 1.61 (1.49-1.73) | 1.50 (1.35-1.65) | 1.19 (0.96-1.48) |
| **CVD mortality** |  |  |  |  |  |  |  |
| Deaths/N | 4932/406 076 | 66/1029 | 162/3313 | 259/5795 | 194/5709 | 83/3251 | 24/1137 |
| Deaths per 1000 person-years | 0.9 | 5.0 | 3.8 | 3.4 | 2.6 | 1.9 | 1.6 |
| HR (95% CI) | 1 (reference) | 3.84 (2.91-5.07) | 2.82 (2.34-3.41) | 2.43 (2.12-2.79) | 1.84 (1.58-2.14) | 1.40 (1.11-1.77) | 1.29 (0.83-2.00) |
| **Cancer mortality** |  |  |  |  |  |  |  |
| Deaths/N | 13 072/406 076 | 71/1029 | 207/3313 | 347/5795 | 313/5709 | 168/3251 | 48/1137 |
| Deaths per 1000 person-years | 2.4 | 5.4 | 4.8 | 4.6 | 4.2 | 3.9 | 3.2 |
| HR (95% CI) | 1 (reference) | 1.83 (1.44-2.32) | 1.57 (1.35-1.82) | 1.42 (1.27-1.59) | 1.29 (1.14-1.45) | 1.22 (1.03-1.45) | 1.05 (0.76-1.45) |

*Hazard ratio was estimated using Cox proportional hazards regression with age as time scale and adjusted for sex, ethnicity, education, and Townsend deprivation index. Imputed exposure and covariates included all risk factors, ethnicity, and education. The estimates from five imputed data set were pooled with the use of Rubin’s rule.

CVD, cardiovascular disease; HR, hazard ratio.

**Table S13. Risk of mortality according to the number of risk factors within target range among participants with type 2 diabetes compared with those without diabetes after excluding deaths within the first 2 years of follow-up**.

|  | **Individuals**  **without diabetes** | **No. of risk factors within target range among individuals with diabetes** | | | | | |
| --- | --- | --- | --- | --- | --- | --- | --- |
|  |  | **0-1** | **2** | **3** | **4** | **5** | **6-7** |
| **All-cause mortality** |  |  |  |  |  |  |  |
| Deaths/N | 17 735/302 063 | 106/586 | 340/2091 | 609/3924 | 556/4096 | 317/2487 | 82/883 |
| Deaths per 1000 person-years | 4.3 | 13.8 | 12.4 | 11.8 | 10.3 | 9.7 | 7.0 |
| HR (95% CI)* | 1 (reference) | 2.64 (2.18-3.20) | 2.14 (1.93-2.39) | 1.88 (1.73-2.04) | 1.62 (1.49-1.77) | 1.53 (1.37-1.71) | 1.16 (0.94-1.44) |
| **CVD mortality** |  |  |  |  |  |  |  |
| Deaths/N | 3195/302 063 | 30/586 | 85/2091 | 167/3924 | 129/4096 | 51/2487 | 19/883 |
| Deaths per 1000 person-years | 0.8 | 3.9 | 3.1 | 3.2 | 2.4 | 1.6 | 1.6 |
| HR (95% CI) | 1 (reference) | 3.85 (2.69-5.52) | 2.77 (2.23-3.44) | 2.64 (2.25-3.08) | 1.94 (1.62-2.31) | 1.28 (0.97-1.68) | 1.40 (0.89-2.20) |
| **Cancer mortality** |  |  |  |  |  |  |  |
| Deaths/N | 9009/302 063 | 39/586 | 127/2091 | 232/3924 | 213/4096 | 122/2487 | 34/883 |
| Deaths per 1000 person-years | 2.2 | 5.1 | 4.6 | 4.5 | 3.9 | 3.7 | 2.9 |
| HR (95% CI) | 1 (reference) | 2.01 (1.47-2.75) | 1.67 (1.40-1.99) | 1.50 (1.32-1.71) | 1.30 (1.14-1.49) | 1.23 (1.03-1.47) | 1.00 (0.71-1.40) |

*Hazard ratio was estimated using Cox proportional hazards regression with age as time scale and adjusted for sex, ethnicity, education, and Townsend deprivation index.

CVD, cardiovascular disease; HR, hazard ratio.

**Table S14. Risk of mortality according to the number of risk factors within target range among participants with diagnosed diabetes compared with those without diabetes**.

|  | **Individuals**  **without diabetes** | **No. of risk factors within target range among individuals with diagnosed diabetes** | | | | | |
| --- | --- | --- | --- | --- | --- | --- | --- |
|  |  | **0-1** | **2** | **3** | **4** | **5** | **6-7** |
| **All-cause mortality** |  |  |  |  |  |  |  |
| Deaths/N | 18 505/302 833 | 73/323 | 274/1536 | 539/3324 | 543/3749 | 322/2359 | 83/862 |
| Deaths per 1000 person-years | 4.5 | 17.9 | 13.7 | 12.5 | 11.0 | 10.4 | 7.3 |
| HR (95% CI)* | 1 (reference) | 3.17 (2.52-3.99) | 2.21 (1.96-2.49) | 1.85 (1.70-2.02) | 1.65 (1.52-1.80) | 1.57 (1.40-1.75) | 1.16 (0.94-1.44) |
| **CVD mortality** |  |  |  |  |  |  |  |
| Deaths/N | 3389/302 833 | 24/323 | 76/1536 | 148/3324 | 130/3749 | 56/2359 | 18/862 |
| Deaths per 1000 person-years | 0.8 | 5.9 | 3.8 | 3.4 | 2.6 | 1.8 | 1.6 |
| HR (95% CI) | 1 (reference) | 5.30 (3.55-7.92) | 3.11 (2.48-3.91) | 2.55 (2.16-3.01) | 2.00 (1.68-2.39) | 1.39 (1.07-1.81) | 1.29 (0.81-2.05) |
| **Cancer mortality** |  |  |  |  |  |  |  |
| Deaths/N | 9396/302 833 | 22/323 | 97/1536 | 200/3324 | 204/3749 | 125/2359 | 36/862 |
| Deaths per 1000 person-years | 2.3 | 5.4 | 4.9 | 4.6 | 4.1 | 4.0 | 3.1 |
| HR (95% CI) | 1 (reference) | 1.96 (1.29-2.98) | 1.64 (1.34-2.00) | 1.44 (1.25-1.66) | 1.30 (1.13-1.50) | 1.27 (1.07-1.52) | 1.04 (0.75-1.45) |

*Hazard ratio was estimated using Cox proportional hazards regression with age as time scale and adjusted for sex, ethnicity, education, and Townsend deprivation index.

CVD, cardiovascular disease; HR, hazard ratio.

**Fig. S1. Flow diagram of the study population**.


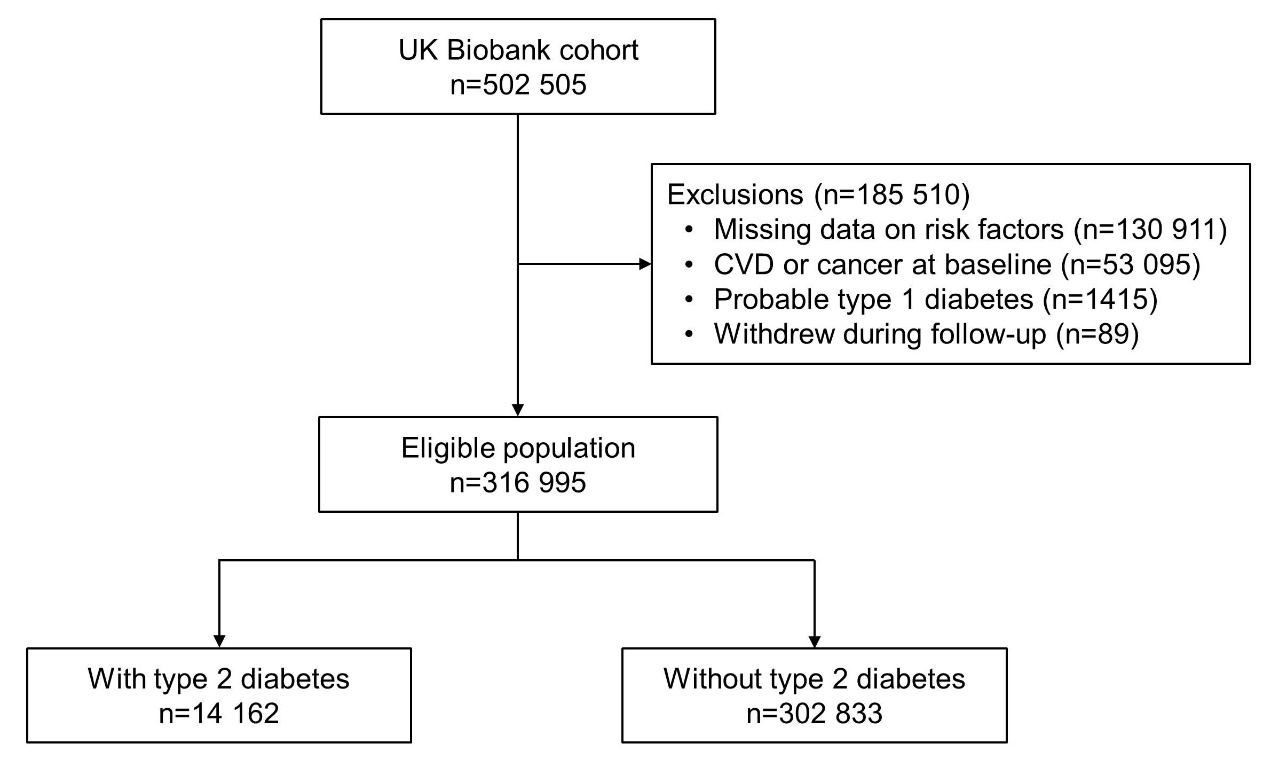


CVD, cardiovascular disease.

**Fig. S2. Number of risk factors within target range among participants without diabetes (A) and participants with type 2 diabetes (B)**.


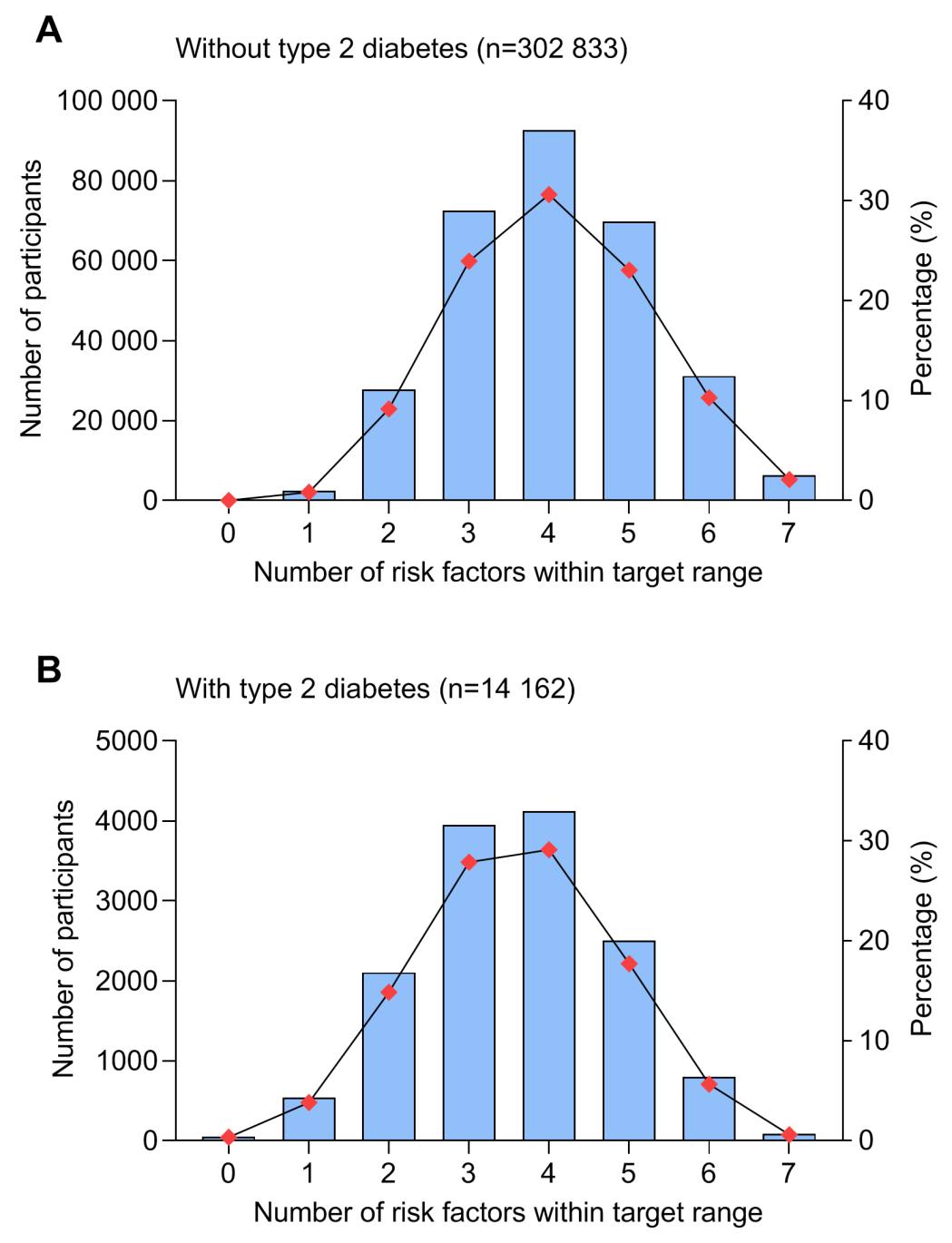


The left y-axis (blue bar) displays the absolute number of participants according to the number of risk factors within target range and the right y-axis (red diamond) displays the percentage of participants.

**Fig. S3. Kaplan–Meier curves for cumulative rate of all-cause mortality (A), CVD mortality (B), and cancer mortality (C) according to the number of risk factors within target range among participants with and without diabetes**.


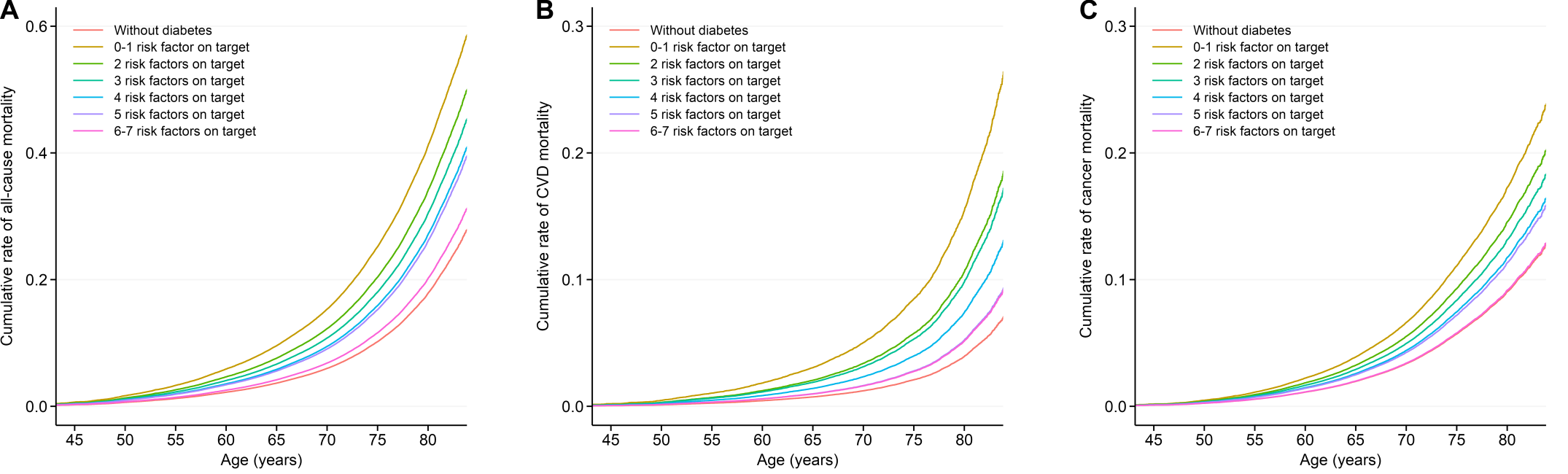


The estimates of cumulative rates were adjusted for age (time scale), sex, ethnicity, education, and Townsend deprivation index.
